# QuantiTrack: A unified software to study protein dynamics in living cells

**DOI:** 10.64898/2026.02.19.706877

**Published:** 2026-02-27

**Authors:** David A Ball, Kaustubh Wagh, Diana A Stavreva, Le Hoang, R Louis Schiltz, Raj Chari, Razi Raziuddin, Davide Mazza, Arpita Upadhyaya, Gordon L Hager, Tatiana S Karpova

**Affiliations:** 1 Laboratory of Receptor Biology and Gene Expression, National Cancer Institute, NIH, Bethesda, MD 20892, USA.; 2 Genome Modification Core, Laboratory Animal Sciences Program, Frederick National Lab for Cancer Research, Frederick, MD 21702, USA.; 3 Università Vita-Salute San Raffaele, Via Olgettina 58, 20132, Milan, Italy.; 4 IRCCS Ospedale San Raffaele, Experimental Imaging Center, Via Olgettina 58, 20132, Milan, Italy.; 6 Department of Physics, University of Maryland, College Park, Maryland 20742, USA.; 7 Institute for Physical Science and Technology, University of Maryland, College Park, Maryland 20742, USA.

## Abstract

Linking the spatiotemporal dynamics of proteins in live cells to physiological functions is a fundamental challenge in biology and robust quantification of protein dynamics is a major step towards this endeavor. Single molecule tracking (SMT) has emerged as a powerful technique to investigate protein dynamics at the single molecule level in living cells. Most SMT analyses require familiarity with biophysical models and programming and the results from different analyses cannot be easily integrated. To mitigate these shortcomings, we developed QuantiTrack – a MATLAB-based SMT analysis software that can be operated from a simple graphical user interface. This provides a much-needed end-to-end solution where a user can load a movie, track single molecules, and perform a range of analyses. In addition to a detailed user guide with step-by-step instructions, QuantiTrack includes quality control metrics that can be used to systematically determine tracking parameters. As a practical example, we address by QuantiTrack a question relevant to hormonal therapy: How does the glucocorticoid receptor (GR), a hormone-regulated transcription factor (TF), respond to treatment and washout of its cognate hormone. Hormone washout results in rapid (in minutes) downregulation of GR target genes to basal levels. We observe dynamics of the Halo tagged GR (Halo-GR) and by integrating several analyses, show that hormone washout results in a substantially lower bound fraction of GR, reduced occupancy in the mobility state associated with GR activation, and shorter GR dwell times. These analyses showcase QuantiTrack as a convenient tool for comprehensive SMT analysis for a wide range of biologists.

## INTRODUCTION

The living cell contains billions of proteins that are constantly moving, interacting with other proteins, nucleic acids, and small molecules to both maintain cellular homeostasis and help the cell respond to external stimuli. Gene regulation offers a particularly striking example of this phenomenon. For instance, early fluorescence recovery after photobleaching (FRAP) experiments showed that TFs rapidly exchange with target sites^1^, challenging the decades-old paradigm that they form stable complexes with co-regulators and chromatin^2^. Shortly thereafter, most nuclear proteins were shown to be highly mobile – binding to their substrates only for short times^3,4^. Today, single molecule tracking (SMT) has become the state-of-the-art technology to study intranuclear protein dynamics across species ranging from yeast^5^, drosophila^6^, and zebrafish^7^, to mammals^8^ because it provides a direct readout of the motion of individual molecules.

For the same set of SMT data, different analytical approaches allow one to extract distinct, complementary information that quantifies the spatiotemporal dynamics of their protein of interest. These include measures of spatial mobility such as mean-squared displacements (MSD) or diffusivity distributions^9–17^, the fraction of bound molecules^18,19^, the anisotropy in spatial mobility^20^, and binding lifetimes (‘dwell’ times)^21,22^. Specifically, those approaches characterize the speed and geometry of the search for target sites, dynamics of free, confined, and bound molecules, and the transitions between bound and diffusive states. The current goal is to connect these parameters to the physiological role of the protein of interest. The success of this endeavor relies on proper data collection and rigorous analysis and interpretation.

Several groups have developed complementary analysis tools that are often used in conjunction with one another. To do this, one must run the same data through a roster of different programs, which creates a bottleneck in the SMT workflow. First, this requires a sophisticated understanding of programming languages along with various biophysical models, something that is not readily available within a molecular biology laboratory. Second, it slows down the quantification, as there is no easy way to transfer the data between programs, and many steps must be repeated in different programs. Finally, comparing similar parameters obtained from different programs and maintaining uniformity therein is challenging.

To overcome this, we developed a comprehensive analysis software called QuantiTrack. This is a MATLAB-based software that runs through a graphical user interface (GUI), where users can identify and track single molecules from a movie, perform quality control (QC), and analyze tens of thousands of single molecule trajectories across hundreds of cells with a few clicks and minimal knowledge of MATLAB. QuantiTrack not only allows the user the freedom to choose between multiple tracking algorithms but also to use well defined QC metrics to systematically determine the tracking parameters. The data are stored in a convenient table format, which serve as the input to perform downstream analyses. Within QuantiTrack, we have bundled the following features that we believe are essential to quantify the spatiotemporal dynamics of a protein-of-interest:

1. Kinetic modeling to calculate fractions of bound and diffusive molecules
2. Iteratively fitting the step size distribution to estimate the distribution of MSDs using the Richardson-Lucy algorithm (RL analysis)
3. Quantifying the anisotropy in the distributions of angular displacements over time and space
4. Fitting ensemble MSD to anomalous diffusion models
5. Measurement of photobleaching rates
6. Calculating photobleaching-corrected dwell time distributions
7. Classifying trajectories into distinct mobility states using perturbation expectation-maximization version 2 (pEMv2)

QuantiTrack comes with a detailed user manual and simple interface, which makes it highly accessible to every molecular biologist, and generates publication quality figures and movies.

In this manuscript, we illustrate the utility of QuantiTrack by using it to study the dynamics of the glucocorticoid receptor (GR, a steroid hormone receptor). GR is a ligand-regulated TF that binds specific DNA sequence motifs within enhancers to regulate target genes. We have demonstrated that the timing and duration of glucocorticoid delivery is an important and often overlooked aspect of treatment protocols^23–25^. Many GR target genes are rapidly activated within minutes of hormone treatment and return to basal levels shortly after hormone washout^23,24,26^. We have shown using FRAP that hormone washout results in faster fluorescence recovery indicating shorter GR binding lifetimes (referred to as ‘dwell times’ in this manuscript), and reduced GR binding via chromatin immunoprecipitation (ChIP)^23^. In this study, we validated these findings using SMT to address how GR dynamics change upon hormone treatment and withdrawal. To examine upstream changes in GR binding dynamics, we used two different imaging protocols: “fast” SMT to capture both bound and diffusing molecules, and “slow” SMT to capture the motion of bound GR molecules and their dwell times^8^. Our data showed that upon hormone washout, the fraction of bound GR molecules (bound fraction) drops by almost 60%. At fast timescales, GR exhibited multiple mobility states, each characterized by their angular anisotropy and MSD power-law scaling exponents. Bound GR presented two low-mobility states, and hormone washout reduced GR occupancy in the lowest mobility state which correlates with the active form of TFs^11,27,28^. Finally, GR dwell times are significantly shorter upon hormone withdrawal. Together, these findings show that a unified SMT analysis platform such as QuantiTrack enables systematic investigation of protein dynamics in cells and clarifies how dynamic changes relate to function, making SMT accessible to a broad community of biologists.

## RESULTS

### QuantiTrack: A GUI-based SMT analysis platform

**Figure 1:**
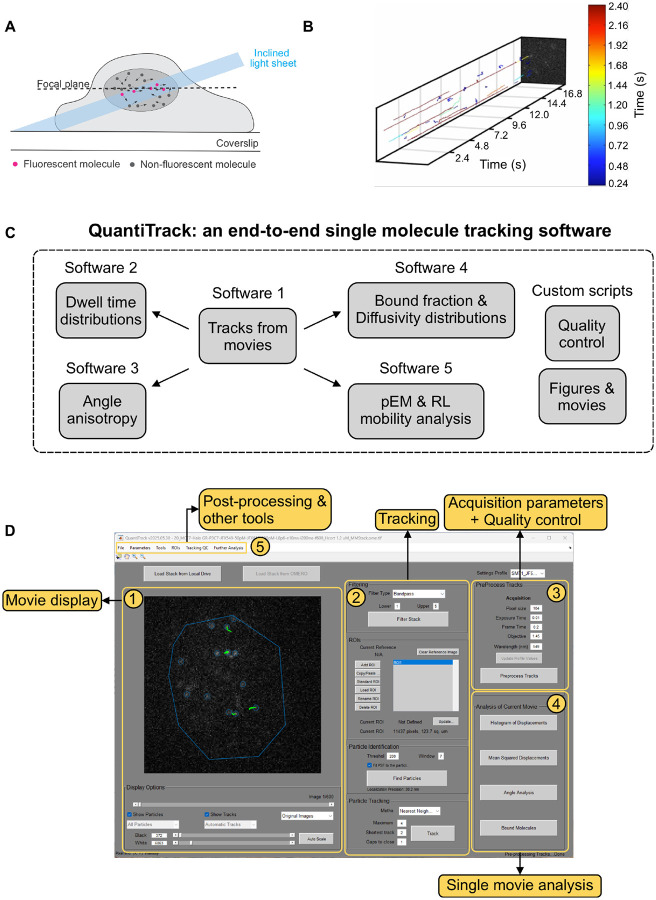
QuantiTrack – a comprehensive GUI based single molecule tracking analysis software. **(A)** Schematic of a single molecule tracking (SMT) experiment. An inclined light sheet is typically used to illuminate a thin section of the cell to minimize background fluorescence. Panel reused with permission from 11 (public domain). **(B)** A (2+1) dimensional snapshot of the output of an SMT experiment. The lines are the detected single molecule trajectories, color-coded according to their track lengths. **(C)** A typical SMT analysis workflow involves the use of multiple software packages to obtain tracks, perform quality control, perform different analyses, and generate movies and publication-quality figures. QuantiTrack provides an end-to-end solution by unifying all these features into a single graphical user interface (GUI)-based software. **(D)** The GUI for QuantiTrack can be divided into five sections: (1) Movie display panel: The movie display panel to view the movie. (2) Tracking panel: To enter tracking parameters, detect particles, and connect particles to form trajectories. (3) Acquisition parameters and quality control panel: The user can set the acquisition parameters and perform quality control for the loaded movie from here. (4) Single movie analysis panel: This panel contains several options of quick analyses for the loaded movie. (5) Post-processing & other tools panel: Additional analyses and tools are available here.

SMT experiments are typically performed by confining the excitation light to a thin sheet (~micrometers for Highly Inclined Laminated Optical [HILO] Sheet microscopy, ~100 nm for Total Internal Reflection Fluorescence [TIRF] microscopy) around the focal plane to reduce out-of-plane fluorescence and achieve high signal-to-noise ratios (SNR) (Figure 1A).

The initial output of an SMT experiment is shown in Figure 1B where single molecule trajectories are represented as kymograms, demonstrating the rich diversity of mobility patterns that may be observed. The typical workflow for SMT analysis involves using a software such as uTrack^29^ or TrackMate^30^ to identify single molecules within the movie and to connect them together into tracks (Figure S1). Multiple software programs are then used to analyze various spatiotemporal characteristics of these trajectories, including but not limited to, dwell time distributions, angle anisotropy, bound fractions, other measures of diffusivity distributions (a non-exhaustive list of software can be found here^31^). Individual research groups must often write custom scripts to perform quality control and to generate publication-worthy figures and movies. The required programming skills are not readily available in every molecular biology laboratory, creating a high barrier-to-entry for a molecular biologist looking to perform SMT. Here, we combine all these features into a GUI-based tool where a user with minimal programming knowledge can perform all these steps with relative ease and obtain quantitative information about their biological system of interest. We call this software QuantiTrack (Figure 1C) and it is based on the original framework of TrackRecord^19^. The QuantiTrack GUI can be divided into 5 panels (Figure 1D).

**Figure 2:**
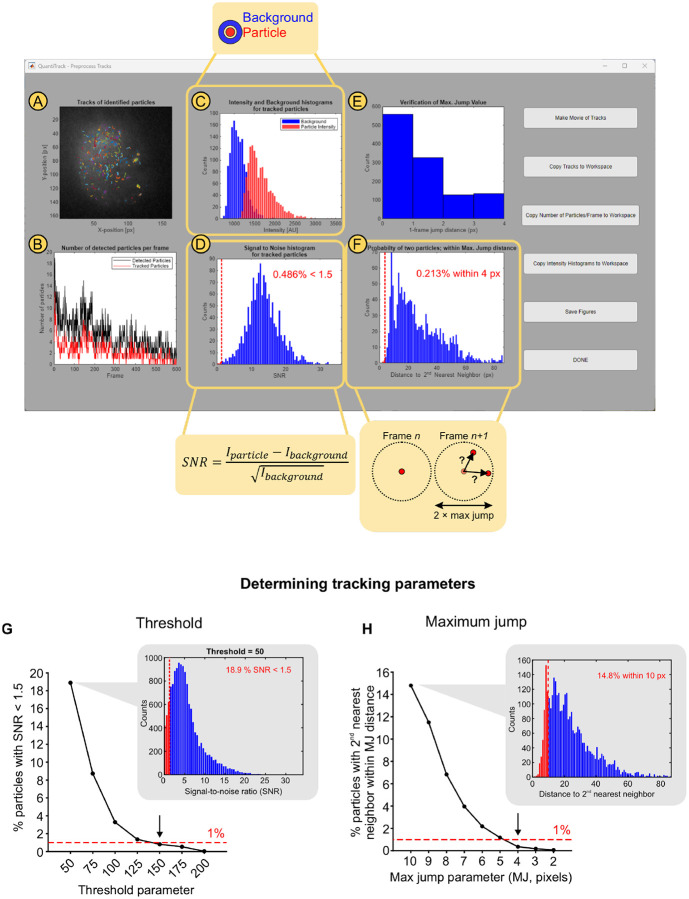
Determining detection and tracking parameters based on quality control metrics. The ‘Preprocess Tracks’ window contains the following qualitative and quantitative quality control measures: **(A)** Tracks overlaid on a temporal projection of the SMT movie. **(B)** Number of detected particles (black) and tracked particles (red) over time, used to assess the fraction of detections incorporated into tracks based on the chosen tracking parameters. **(C)** Histograms of particle (red) and local background (blue) intensities. (Callout) Particle intensity is calculated as the summed intensity of the Airy disk, while the local background is estimated from a surrounding donut region. **(D)** Histogram of signal-to-noise ratios (SNR) for all detected particles. Bins with SNR < 1.5 are shown in red to the left of the dashed red line. The inset reports the percentage of particles with SNR < 1.5. (Callout) SNR calculation formula. **(E)** Histogram of single frame displacements (in pixels). **(F**) Histogram of distances between a tracked particle in frame n and its second nearest neighbor in frame n+1. (Callout) Tracking errors can arise if multiple particles fall within the maximum jump radius (MJ). Distances ≤ MJ are shown in red to the left of the dashed red line. The inset reports the corresponding percentage. **(G)** Percentage of particles with SNR < 1.5 as a function of the Threshold parameter. The callout shows the SNR histogram for Threshold = 50. See also, panel D. We recommend selecting the Threshold using an objective cutoff for the fraction of low-SNR detections (here, 1%; red dashed line), yielding Threshold = 150 (arrow). **(H)** Percentage of second nearest neighbors within MJ as a function of the MJ parameter. The callout shows the histogram of such distances for MJ = 10 pixels (px). See also, panel F. We suggest choosing the max jump where this percentage falls below 1% (red da shed line). MJ = 4 pixels satisfies this criterion (arrow).

1. Movie display panel: Here, a user can load in their single molecule movie, adjust visualization parameters and toggle through the entire movie.
2. Tracking panel: The user can select various parameters related to filtering the movie, defining the region of interest (ROI) within the field of view where the particles should be detected, defining particle detection parameters, and methods of particle tracking.
3. Acquisition parameters and quality control panel: Through this panel, the user sets the data acquisition parameters and accesses the QC window through the ‘Preprocess Tracks’ button (see user guide and Figure 2 for more details).
4. Single movie analysis panel: This offers four types of analyses for the currently loaded movie.
5. Post-processing & other tools panel: These panels allow the user to perform the analyses shown in Figure 1C over all the movies in their datasets.

The typical workflow for tracking a single movie in QuantiTrack involves loading the movie into the GUI, filtering the movie, drawing the ROI, finding particles, and iteratively tracking and performing quality control until certain QC thresholds have been met (Figure S1). The next section describes QC metrics available within QuantiTrack, and how to use these to determine particle detection and tracking parameters.

### On the fly quality control measures built into QuantiTrack

Before analyzing SMT movies, we recommend that the movies be visually inspected to check for cell-scale motion. Any movies with visible translation or rotation should be discarded before tracking. For movies that pass this initial step, QuantiTrack offers several QC and visualization options.

Figure 2A–F shows a representative screenshot of the pop-up window that appears after clicking the ‘Preprocess Tracks’ button in the main QuantiTrack GUI (Figure 1D, **panel 3**). This window allows the user to inspect the quality of particle detection and tracking and change any parameters if needed. The first panel (Figure 2A) contains an overlay of all the tracks over a time-projection of the SMT movie, allowing the user to spot erroneous tracks. Figure 2B shows the number of detected particles (black) and the number of tracked particles (red), enabling the user to gauge whether most of the detected particles are being tracked with the current settings. Some of the detected particles may not be tracked – for example, particles that produce tracks shorter than the shortest track parameter will not be recorded as tracks.

The panels in Figure 2C–F show two quantitative measures to estimate the frequency of tracking errors. Two common sources of erroneous particle tracking are: (1) incorrectly detected particles and (2) mistracking (i.e. connecting distinct nearby particles into a single track). QuantiTrack enables the user to estimate these errors for each movie.

The first issue typically occurs when background is mistakenly detected as a particle. To quantify this, we calculate the particle intensity and the local background intensity for each detected particle (Figure 2C). The particle intensity is calculated from the total intensity within the Airy disk surrounding the particle coordinates, and the local background is calculated from a donut shaped ROI around this Airy disk (Figure 2C and D, callouts). Figure 2C shows an example of the signal (particle intensity) and noise (background) histograms when the particle detection is robust. QuantiTrack also measures the SNR for all the detected particles and calculates the fraction of particles with an SNR lower than 1.5 (see Methods). An objective cutoff for the fraction of particles with SNR < 1.5 can be used to determine the appropriate threshold value. We recommend using a cutoff of 1–5%.

The Threshold parameter (Figure 1D, **panel 2**) determines which particles are detected. A low Threshold value will result in false positives (background detected as particles) while a high Threshold value will miss some of the dimmer particles. The SNR histogram (Figure 2D) can be used to set this threshold as follows:

1. Perform a parameter sweep of the threshold values.
2. For each threshold, record the percentage of particles with SNR < 1.5.
3. Use the threshold value at which less than the empirical threshold (1% in this example) of particles has an SNR < 1.5.

Figure 2G illustrates this approach. We ran the particle detection step with Thresholds ranging from 50 to 200 in steps of 25. The callout in Figure 2G shows the SNR histogram for Threshold = 50, in which ~19% of particles have an SNR < 1.5. As the threshold is increased, this number drops and first falls below 1% for Threshold = 150 (Figure 2G and S2). This process can now be repeated with a narrower parameter range (say 125 to 175) for other movies, assuming that the labeling density and/or expression level are similar. In our experience, the same threshold is typically sufficient for movies with comparable labeling densities.

Figure 2E shows a histogram of inter-frame displacements which confirms that most of the displacements are well within the allowed maximum jump ([MJ], set to 4 pixels or 416 nm in this example).

While this serves as a qualitative assessment of tracking errors arising due to crowding or incorrect tracking parameters, QuantiTrack also provides a quantitative measure of the error rate. Mistracking errors occur when a particle detected in one frame is incorrectly linked to a different particle in the subsequent frame. Such errors would occur if, for a particle in frame n, two particles are present within the maximum jump radius in frame n+1 (Figure 2F, callout). This can be quantified by measuring the distance between every tracked particle and its second nearest neighbor in the subsequent frame (see Methods). From the histogram of all such distances, QuantiTrack calculates the fraction of second nearest neighbors which fall within the maximum jump radius. The percentage of second nearest neighbor distances within the maximum jump radius is shown in the inset (Figure 2F, inset). Care should be taken to keep the particle labeling density is low enough that this percentage stays small.

A parameter sweep can also be used to determine the optimal MJ. Here, the fraction of particles with second nearest neighbors within MJ in the subsequent frame can be used as a metric to determine the appropriate MJ parameter. A range of MJ parameter values can be tested and the one at which percentage of particles with second nearest neighbors within the MJ distance falls below the defined cutoff (1–5%) should be selected. As shown in Figure 2H (callout), max jump = 10 pixels results in ~15% likelihood of mistracking. Reducing the max jump in steps of 1 pixel reduces the probability of mistracking and this falls under 1% for MJ = 4 (Figures 2H and S3).

The particle and background intensities, and second nearest neighbor distances are saved in the track-Table that is generated by QuantiTrack (see user guide) and can be used for diagnostic purposes. To maintain consistency, we recommend that for every new protein and cell line combination, these parameters be optimized on a set of movies, and kept consistent from one movie to another for the same protein, cell line, dye, and treatment combination, so long as the acquisition parameters remain the same.

Additionally, the buttons to the right of the figure windows (Figure 2A–F) contain options to:

1. Generate a movie (see user guide for details), with the option of rendering it in (2+1) D with time on the third axis (Movie 1, Figure 1B) or in 2D (Movies 2 and 3).
2. Copy the tracks (Figure 2A), number of detected and tracked particles (Figure 2B), and intensity histograms (Figure 2C) to the MATLAB workspace for further analysis.
3. Save the plots displayed in the Preprocessing window.

We should note that these recommendations do not imply that tracking parameters can be adjusted to fix issues with labeling density, laser powers, or acquisition parameters. For example, if the signal intensity from molecules is too low (because the exposure time is too short, laser power not high enough, or too high a background), increasing the Threshold will result in the detection of only the brightest particles. However, this could result in long tracks being chopped up into short discontinuous tracks, that could, for instance, provide completely incorrect estimates of the binding kinetics. Similarly, if the labelling is very dense, one can reduce the maximum jump such that mistracking is minimized, but this would result in artificially constrained tracks which would affect measurements of spatial mobility such as jump distances or MSD analyses. These issues can be mitigated in the early stages of designing SMT experiments by inspecting the movies of tracks overlaid on the raw images, using the ‘Make Movie of Tracks’ button in the preprocessing step (Figure 2).

To demonstrate the value of QuantiTrack, we next applied it to quantitatively study GR dynamics under conditions of hormone treatment and washout.

### Glucocorticoid receptor target genes and GR dynamics respond to hormone treatment and washout

**Figure 3:**
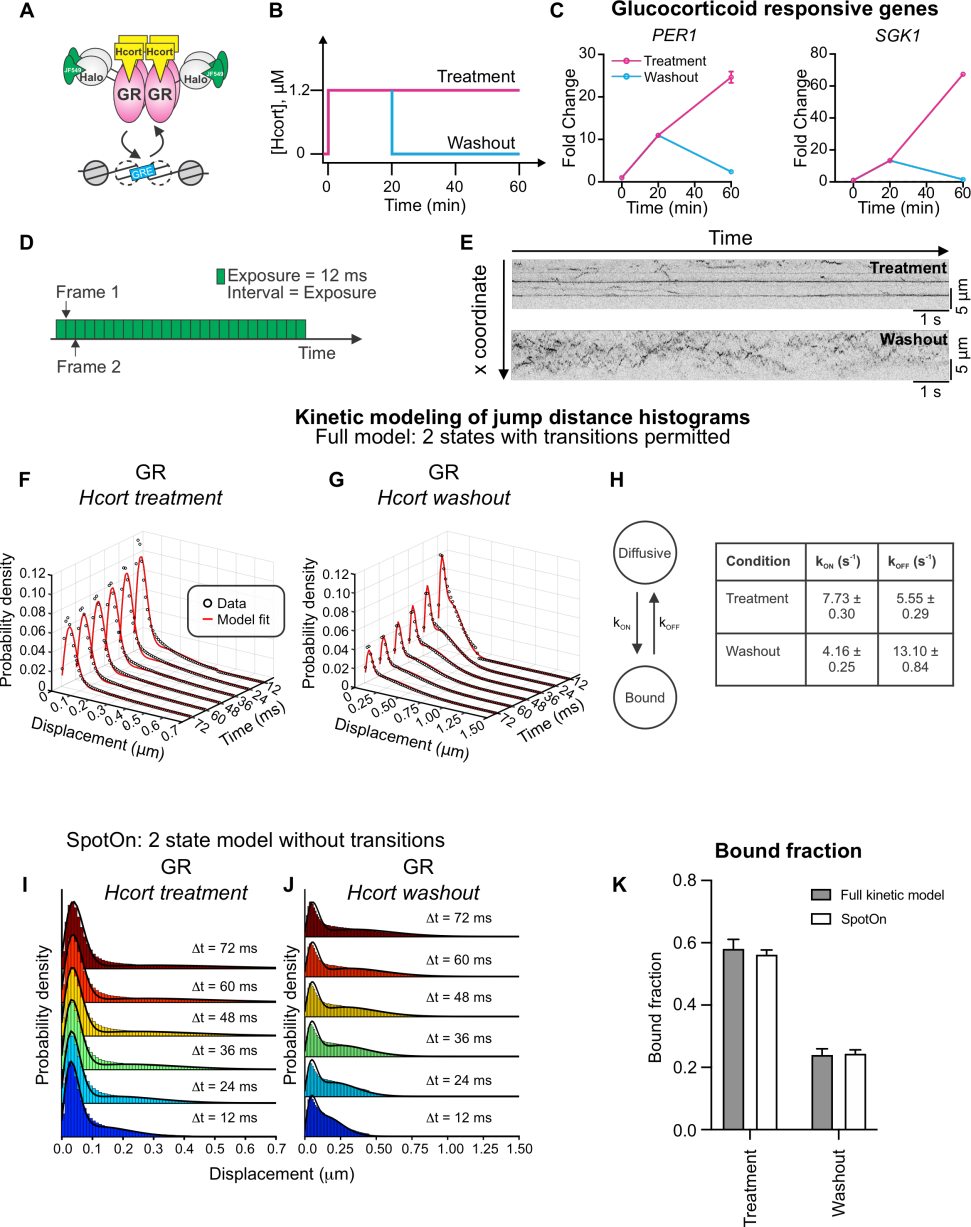
Hormone washout leads to downregulation of GR-responsive genes and significantly reduces GR bound fraction. **(A)** Activated GR dynamically binds to glucocorticoid response elements (GREs) as a tetramer. **(B)** Schematic of hormone treatment and washout protocol. Hcort = hydrocortisone, a natural glucocorticoid. **(C)** qPCR data for two GR responsive genes (PER1 and SGK1) under Hcort treatment and washout at the indicated time points. Error bars = standard error of the mean. All data are normalized to the vehicle control. **(D)** Schematic of the imaging protocol for fast SMT. Images are collected every 12 ms with continuous exposure for 1500 frames. **(E)** Representative kymograms of GR under Hcort treatment (top) and Hcort washout (bottom). Stripes indicate bound molecules. **(F–G)** Kinetic modeling of jump distance histograms over six time lags using the full kinetic model with two states (bound + diffusive) for GR under (F) Hcort treatment and (G) Hcort washout. The data are shown as black circles and the model fit is in red. **(H)** The full kinetic model allows for transitions between the bound and diffusive state. kON and kOFF denote the transition rate from diffusive to bound and vice versa. The table shows the transition rates as calculated by the model. **(I–J)** Jump displacement histograms and accompanying model fits obtained from Spot-On. The solid lines denote the fits to a two-state model. **(K)** Bound fraction calculated from the full kinetic model (gray) and Spot-On (white) for GR under Hcort treatment and washout. Error bars = bootstrapped standard deviation. Ncells/Ntracks = 130/93,836 (GR + Hcort treatment), 133/117,085 (GR + Hcort washout).

GR is activated upon the binding of its cognate ligands (glucocorticoids [GC]) and serves as a TF that regulates the stress response, inflammation, metabolism, cognitive function, and immune response, among others^32^. GR is primarily cytoplasmic in the absence of hormone, sequestered by a chaperone complex. Upon ligand binding, GR translocates to the nucleus and remains nuclear after hormone washout^23,33^. When conjugated to an agonist, GR dynamically binds glucocorticoid response elements within enhancers as a tetramer^34^ (Figure 3A).

In mammals, GCs are secreted in circadian rhythms, with the peak in hormone release coinciding with the active phase of the animal^35^. Underlying this circadian pattern, are short, roughly hourly, ‘ultradian’ pulses of glucocorticoid release^36^. Work from several labs, including ours, has studied the effect of pulsatile hormone release on GR-responsive gene expression^23,24,26,37^. In response to ultradian GC pulses, many GR-target genes are also transcribed in a pulsatile manner^23,24,26,37,38^. The mechanism behind pulsatile transcription has been linked to differential recruitment of GR to GREs^23^ and enhancer-specific changes in accessibility^26^. FRAP experiments showed that compared to constant hormone exposure, GFP-GR fluorescence recovery at a tandem array of GR binding sites is faster upon hormone washout^23^. However, FRAP represents an ensemble measurement, with diffusing molecules providing a major contribution to the FRAP curves^39^. No detailed single molecule characterization of GR dynamics under hormone treatment and washout conditions has been performed. Here, we use this well-defined system to show how QuantiTrack enables quantitative, single-molecule dissection of GR binding dynamics upon hormone stimulation and washout.

To simulate the effects of hormone pulses, we expose cells to a 20 min pulse of hydrocortisone (Hcort), which is the pharmaceutical name of the natural human GC cortisol. Following this pulse, we either maintain the cells in Hcort-containing medium, which we refer to as the ‘treatment’ condition in this manuscript, or washout the hormone using conditioned medium (Figure 3B, see Methods). The latter protocol is referred to as the ‘washout’ condition hereon.

We tested the effect of hormone withdrawal in MCF-7 cells on two GR target genes – *PER1* and *SGK1* – and found that both genes are robustly activated 20 min after hormone treatment and exhibit even higher activation at 60 min under the treatment protocol. On the other hand, upon hormone washout, both *PER1* and *SGK1* return to their baseline expression at 60 min (Figure 3C). This is in line with previous studies in mouse cells as well as in animal models^23,24,38^.

Based on the FRAP data^23^, changes in GR target gene expression likely reflect upstream changes in GR dynamics. To study this, we generated a clonal MCF-7 cell line with a HaloTag knocked in at the endogenous *NR3C1* locus (which encodes the GR protein) using CRISPR-Cas9 (Figure S4A–B, see Methods). We also generated an MCF-7 cell line expressing H2B-Halo from the endogenous H2B promoter (Figure S4C, see Methods). As expected, Halo-GR exhibits predominantly cytoplasmic localization in the absence of hormone, and is primarily nuclear upon hormone treatment, while H2B-Halo is nuclear and correlates with DAPI staining (Figures S4D and E).

We first determined the fraction of bound GR molecules in the nucleus. To capture both bound and diffusing molecules, we collected images of Halo-GR under Hcort treatment and washout conditions every 12 ms under continuous illumination (Figure 3D). We performed the QC steps outlined above, ensuring that both the SNR and the tracking error rate are under 5% for GR under both Hcort treatment and washout (Figure S5). Representative kymograms show the stark difference between GR dynamics under hormone treatment and washout, with long stripes visible in the treatment condition but only short, highly dynamic trajectories visible in the washout condition (Figure 3E, and Movie 2).

QuantiTrack provides both a kinetic modeling method developed by Mazza et al.^19^ and Spot-On, developed by Hansen et al.^9^ to estimate the bound fraction of the protein of interest from the jump distance histograms across multiple lag times. The key difference between the full kinetic model and Spot-On is that the kinetic model accounts for transitions between the different states while Spot-On does not. As a result, Spot-On is more computationally efficient. Since the transition rates may not always be pertinent to the research question, we have included both the kinetic model and Spot-On in QuantiTrack.

We first benchmarked both methods using publicly available data kindly provided by Anders Hansen and colleagues^40^, and compared the results from the kinetic model with those reported using Spot-On^9^ in the original publication^40^. This study examined the mobility of wildtype (WT) and mutant CTCF, an architectural protein containing 11 zinc fingers (ZF) and an internal RNA-binding region (RBR_i_). They quantified the bound fraction of Halo-mCTCF-WT (mCTCF is mouse CTCF) and a CTCF mutant lacking the RBR_i_ (ΔRBR_i_-Halo-mCTCF), one with His-to-Arg mutations in all the 11 ZFs (11ZF-mut-Halo-mCTCF), and a mutant with all the ZFs deleted (ΔZF-Halo-mCTCF) (Figure S6A) in mouse embryonic stem cells (mESC)^40^. For consistency with analysis performed in the original publication^40^, we restricted our analysis to a two-state model (with one bound state and one diffusive state) with 4 time lags. The jump distance histograms from the kinetic model show that mutations in the RBRi or ZFs broadens the displacement histograms (Figures S6B–F) and result in a reduction in the bound fraction of CTCF (Figure S6G). Notably, the results from the kinetic model are remarkably consistent with those from Spot-On (Figure S6G) with the two endogenous Halo-mCTCF knock-in clones (C59 and C87) showing the highest bound fractions, followed by ΔRBR_i_-mCTCF, 11ZF-mut-mCTCF, and ΔZF-mCTCF respectively. This analysis shows that the QuantiTrack can robustly identify diffusive and bound states of SMT trajectories.

We next used this kinetic model to obtain transition rates between the bound and diffusive states of GR under both conditions. We used both the kinetic model and Spot-On to fit a two-state model to the GR jump distance histograms for both treatment and washout conditions across 6 lag times (simultaneously) (Figure 3F–K, see Methods). The data showed that hormone washout significantly reduced the GR bound fraction by almost 60%, from ~58% (Hcort treatment) to ~24% (Hcort washout) (Figure 3K), as expected. While the bound fractions calculated from both the kinetic model and Spot-On are remarkably consistent (Figure 3K), the kinetic model also showed that the reduction in GR bound fraction is the result of a ~50% reduction in k_ON_ along with a ~2.4-fold increase in k_OFF_ (Figure 3H). Here, k_ON_ is the transition rate from the diffusive state to the bound state, while k_OFF_ is the transition rate from the bound state to the diffusive state.

We next investigated how GR scans the nuclear space in the presence and absence of hormone.

### Identifying distinct mobility modes from fast SMT data

**Figure 4:**
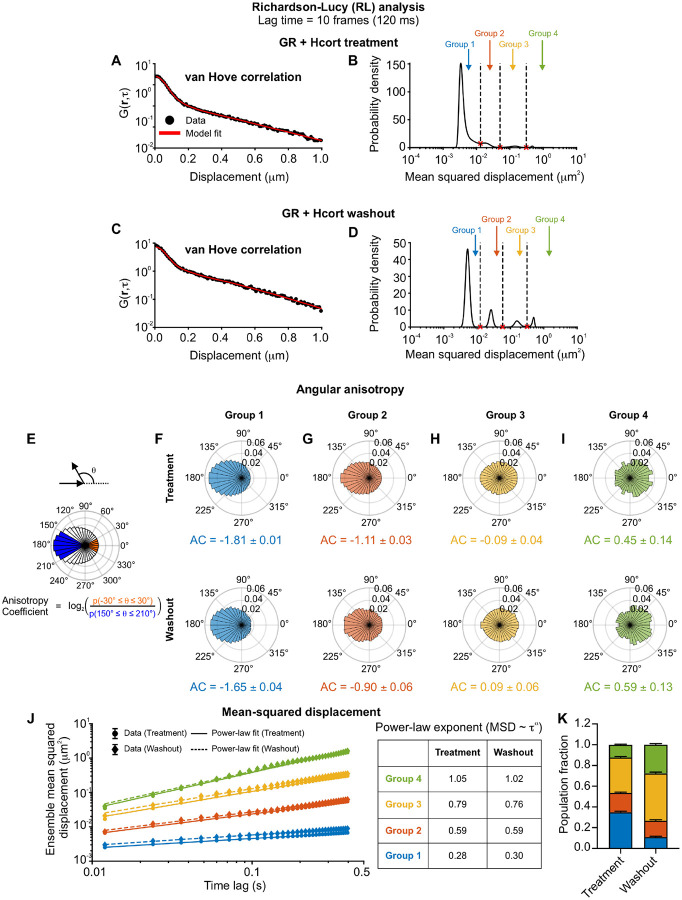
Identifying distinct mobility states from fast SMT data. **(A, C)** van Hove correlation at a lag time of 120 ms (black circles) and model fit obtained using the iterative Richardson-Lucy (RL) algorithm for (A) GR + hydrocortisone (Hcort) treatment and (C) GR + Hcort washout. **(B, D)** Mean squared displacement (MSD) distributions showing multiple mobility groups obtained from the RL analysis for (B) GR + Hcort treatment and (D) washout. Local minima (red crosses) are used to bin the distribution into 4 groups. **(E)** (Top) The angular displacement θ is the angle between two displacement vectors. (Center) Polar histogram of angular displacements with forward displacements indicated in orange and backward displacements in blue. (Bottom) Anisotropy coefficient (AC) is calculated as the log_2_ ratio of the probabilities of forward (orange) to backward (blue) jumps. **(F–I)** Single frame polar histograms of angular displacements for each of the indicated RL groups. (Top) GR + Hcort treatment, (Bottom) GR + Hcort washout. The calculated AC is shown below each polar histogram. The errors represent bootstrapped standard deviations. **(J)** (Left) The ensemble mean-squared displacement for GR after (circles) Hcort treatment and (diamonds) washout for each RL group. Power-law fits are shown as solid lines for GR + Hcort treatment and as dashed lines for GR + Hcort washout. Error bars denote s.e.m. (Right) Table of power-law exponents for the indicated datasets. **(K)** Population fractions for each of the RL groups for the indicated conditions. Error bars = bootstrapped standard deviation. N_tracks_ for GR + Hcort treatment: 2,988 (group 1), 1,594 (group 2), 2,920 (group 3), 1,059 (group 4). N_tracks_ for GR + Hcort washout: 690 (group 1), 946 (group 2), 2,738 (group 3), 1,663 (group 4).

Kinetic modeling is useful to quantify diffusion co-efficients and transition rates when the number of states is known. However, this is often precisely the information that needs to be deduced from the data. QuantiTrack contains a method to estimate the distribution of MSDs (or equivalently, the distribution of diffusion coefficients) without *a priori* information about the number of states. This method is based on an iterative scheme inspired by the Richardson-Lucy algorithm for image processing, and was originally developed by Steve Granick and colleagues^41^, and later adapted by Masaki Sasai and colleagues for quantifying histone dynamics^13^. This method is referred to as ‘RL analysis’ in QuantiTrack (see User Guide, Methods). We used this method to estimate the MSD distribution from the van Hove correlation (vHc) or jump distance histogram as has been done previously^11,13^ (Figure 4A–D). We performed RL analysis on GR trajectories upon Hcort treatment and washout and found that GR exhibits four distinct mobility groups (corresponding to peaks in the MSD histogram) under both conditions (Figure 4A–D). The minima in the MSD histograms can be used to classify tracks into four mobility groups, as has been done previously^11,28^ (Figure 4B, D; see Methods). The tracks belonging to these four groups exhibit progressively larger exploration areas, consistent with their increasing MSDs (Figure S7A–B).

Each of these mobility groups can be characterized by the ensemble angular anisotropy and MSD. The angular anisotropy coefficient (AC) is defined as the log_2_ ratio of the probability of observing a forward jump to that of observing a backward jump^20^ (Figure 4E). Variations of this metric have been previously described, for example in^40,42^. The angular displacement θ is defined as the angle between two displacement vectors and forward jumps are any angles -30°≤θ≤30° while backward jumps are angles such that 150°≤θ≤210°. Lower AC values indicate more confined tracks, while pure Brownian motion corresponds to AC = 0.

Groups 1 and 2 present the most negative AC indicating that these represent two different confined states (Figure 4F–G), while Groups 3 and 4 have AC close to zero, suggesting that these are likely diffusive groups (Figure 4H–I). We also calculated the spatial anisotropy across all four groups, defined as AC as a function of the average displacement (see Methods), and found that the trend of increasing AC from groups 1 to 4 also held up across displacements of 50 to 400 nm (Figure S7C–D).

The ensemble MSD as a function of time lags typically scales as a power-law $\left(\text{MSD}\sim \tau^{\alpha }\right)$, with the power-law exponent α characterizing the nature of the motion. For example, sub-diffusive motion has α<1, while Brownian motion has a scaling exponent α=1. Groups 1–3 exhibit sub-diffusive scaling with α≈0.3,0.6,0.8 for groups 1,2, and 3 respectively (Figure 4J and S8). Group 4 has a scaling exponent of 1, representative of Brownian motion (Figure 4J and S8).

The AC values and MSD power-law exponents are not substantially different between the treatment and washout conditions, indicating that GR under both conditions exhibits the same four mobility groups (Figure 4F–J). However, the fraction of tracks in the lower mobility group (Group 1) reduced from ~35% in the treatment cohort to only ~11% upon hormone washout. The population fraction of the diffusive group (Group 4) increased from ~12% (treatment) to ~28% (washout) (Figure 4K).

While further simulations will help interpret the biological significance of these mobility groups, this analysis demonstrates an unbiased way to distinguish distinct modes of motion. However, although fast SMT allows us to track both bound and diffusive molecules, photobleaching prevents the observation of binding events lasting on the order of seconds. To overcome this limitation, we next modified our imaging protocol to quantify the long-timescale spatiotemporal dynamics of bound GR molecules.

### Hormone washout results in shorter GR dwell times and reduced occupancy of mobility states correlated with transcriptional activity

**Figure 5:**
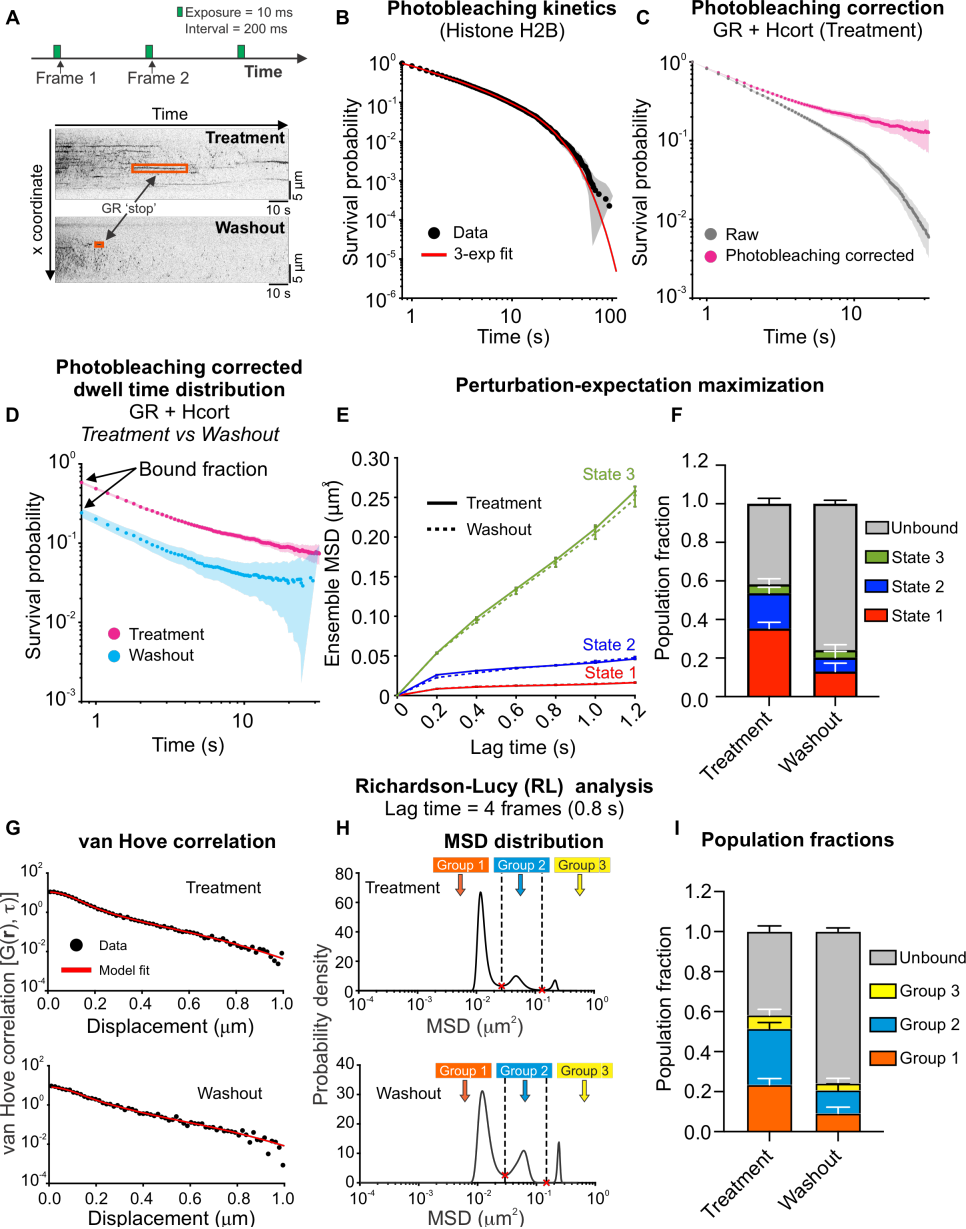
Hormone washout reduces GR dwell times and occupancy of the transcriptionally active mobility state. **(A)** (Top) Imaging protocol for slow SMT. Images are acquired every 200 ms with an exposure time of 10 ms for 2 min (600 frames). (Bottom) Representative kymographs of GR under hydrocortisone (Hcort) treatment (top) and washout (bottom) conditions. Stationary GR molecules appear as stripes (orange boxes). **(B)** Histone H2B survival distribution is used to calculate the photobleaching rate. The raw H2B survival distribution (black) is fit to a triple exponential function (red), and the slowest exponential component defines the photobleaching rate. Shaded error bars indicate 95% confidence intervals (CI). **(C)** Survival distributions for GR under Hcort treatment, before (gray) and after (magenta) correcting for photobleaching. Shaded error bars = 95% CI. **(D)** Photobleaching-corrected survival distributions normalized to the bound fraction for GR + Hcort treatment (magenta) and washout (cyan). Shaded error bars = 95% CI. N_cells_/N_tracks_ = 42/33,173 (H2B), 96/58,239 (GR + Hcort treatment), 70/32,183 (GR + Hcort washout). **(E)** Ensemble mean-squared displacements (MSD) for three mobility states identified using perturbation-expectation maximization (pEMv2). Solid lines: Hcort treatment, dashed lines: Hcort washout. Error bars = s.e.m. N_sub-tracks_ = 18,811 (GR + Hcort treatment), 3,971 (GR + Hcort washout). **(F)** Population fractions of the three pEMv2 states, normalized to the bound fraction. Red: state 1, blue: state, green: state 3, gray: unbound fraction. Error bars = bootstrapped standard deviation. **(G)** van Hove correlation for GR + Hcort treatment (top) and washout (bottom). Black circles: data, red: model fit. **(H)** MSD distribution from the RL analysis for GR + Hcort treatment (top) and washout (bottom). The minima in the MSD distribution are used to classify tracks into different mobility groups. After RL classification: Ntracks for GR + Hcort treatment: 2,475 (group 1), 2,962 (group 2), 716 (group 3). Ntracks for GR + Hcort washout: 561 (group 1), 711 (group 2), 209 (group 3). **(I)** Normalized fractions of tracks in RL Groups 1 (orange), 2 (sky blue), 3 (yellow), and unbound (gray) with bootstrapped standard deviation.

To examine the dynamics of bound GR molecules, we modified our imaging protocol as follows: images were collected with 10 ms exposure times (to minimize motion blur) and an interval of 200 ms (to ensure that fast diffusing molecules are not imaged), for a total duration of 2 min (Figure 5A, Movie 3). This slow SMT protocol allows us to specifically capture the motion of bound molecules. By separating the excitation pulses by 200 ms, we both minimize the probability of capturing diffusing molecules and increase the likelihood of tracking bound molecules over tens of seconds. The binding events of GR molecules are visible as ‘stops’ that appear as stripes on a kymogram (Figure 5A) for both hormone treatment and washout. The dwell time distribution of these ‘stopped’ events provides a measure of the binding times of TFs. To quantify the temporal behavior of such bound molecules, we must account for photobleaching, which would otherwise result in the underestimation of the true dwell time distribution: molecules that remain in the focal volume longer than photobleaching permits will disappear not because they leave the focal volume, but due to photobleaching. We refer the reader to a recent publication for a detailed discussion on the concept of photobleaching^6^. A stable protein such as histone H2B has been used as a probe for photobleaching in the context of nuclear proteins^21,43^. We therefore collected H2B-Halo data, using the same imaging protocol and acquisition parameters, as a control for photobleaching.

The merged SNR histograms for H2B-Halo and Halo-GR show that all tracked particles have high SNRs under all conditions (Figure S9A–C). The tracking error rate was also well under 1% for H2B and GR (both treatment and washout) (Figure S9D–F).

We first calculated the survival distribution of H2B, which is an essential step to accurately measure dwell time distributions of TFs. The survival distribution is the probability of observing a binding event longer than a certain time. Formally, the survival distribution is calculated as S(t)=1-CDF(t), where the CDF is the cumulative distribution function. We fit the histone H 2 B survival distribution to a triple exponential function to estimate the photobleaching rate (Figure 5B) as has been described previously^11,21,27,44^ (see Methods). The photobleaching rate calculated from the H2B data was used to perform the photobleaching correction on the raw GR survival distribution (Figure 5C). Further, the y-intercept of the survival distribution must be normalized to the bound fraction (obtained from the kinetic model; Figure 3K) since the slow SMT protocol only tracks bound molecules. The data showed that hormone washout significantly reduces the binding lifetimes of GR, corroborating our previously published FRAP data^23^ (Figure 5D).

A single TF ‘stop’ that appears as a stripe in a kymogram (Figure 5A) is not necessarily a site-specific binding event. Within a single kymogram stripe, the TF molecule engages in different kinds of motion (we refer the reader to^8^ for a detailed discussion on this). We have previously demonstrated that, on a time scale of 1.2 s, TFs exhibit primarily two distinct mobility states, which are related to the motion of the chromatin fiber^11,27^. Upon activation with their respective hormones, steroid hormone receptors predominantly bind in the lowest mobility state (which we refer to here as state 1) and binding in this state requires an intact DNA-binding domain as well as domains necessary for the recruitment of transcriptional cofactors^11^.

QuantiTrack provides an algorithm called perturbation expectation maximization v2, developed by Simon Mochrie and colleagues,^45^ (hereon referred to as pEMv2) to classify the diffusive motion of SMT trajectories, taking into account localization errors and motion blur artifacts inherent to single molecule tracking in a model-agnostic manner (i.e. whether the particles exhibit free or anomalous diffusion) (Figure S10A). The method ascribes the motion of particles to several potential diffusive states (between 1 and 15) and uses a Bayesian information criterion to determine the optimal number of states that best describes the collection of trajectories. Given the difficulty in inverting large co-variance matrices of the diffusive trajectories, it is optimal to split long trajectories into smaller sub-tracks. Each sub-track is then assigned to the state for which it has the maximum posterior probability (Figure S10A). We have empirically determined that 7 frame long sub-tracks provide a balance between calculation overhead and accuracy^27^.

We applied pEMv2 to GR trajectories under both hormone treatment and washout conditions and found that GR exhibits 3 distinct mobility states: state 1 has the lowest mobility followed by states 2 and 3 (Figures 5E, S10B–E). After normalizing the pEMv2 fractions to the bound fraction (see Methods), we found that hormone washout results in an ~63% reduction in the fractions of state 1 and state 2 (Figure 5F), suggesting that GR cannot bind effectively in the transcriptionally competent state in the absence of hormone, consistent with previous work using transcriptionally inactive TF mutants^11,27^.

Since pEMv2 operates on sub-tracks, we also applied RL analysis to the slow SMT data in order to examine the mobility of entire tracks. As we have described previously for GR and other nuclear receptors, RL analysis reveals 3 to 4 distinct mobility groups at a lag time of 0.8 s for both hormone treated and hormone washed out GR molecules^11^ (Figures 5G). Note that we use the term ‘groups’ for the RL groups to avoid confusion with the pEMv2 ‘states’ described above. As before, we used the minima in the MSD distribution to classify the tracks into different mobility groups (Figure 5H) and found that hormone washout results in a significant reduction in the fraction of GR tracks in group 1, which is the lowest mobility group (Figure 5I), which has been shown to correlate with a reduction in transcriptional response^28^.

Together, these data show that upon hormone washout, GR bound fraction, dwell times, and the fraction of molecules in the mobility state correlated with transcriptional activation (state 1) drops significantly, which results in the downregulation of GR target genes (Figures 3 and 5).

## DISCUSSION

### QuantiTrack: a single GUI to do it all

Single molecule tracking has become a leading approach to study protein dynamics and interactions in living cells. Over the past two decades, SMT has been applied to diverse systems – membrane proteins, molecular motors, nuclear and nucleolar factors – and has also been used to infer rheological properties of the cellular sub-compartments^31^. Most of this work has been driven by the biophysics community and has remained peripheral to mainstream molecular biology.

The basic building blocks of an SMT experiment are a HILO, TIRF, or light sheet microscope equipped with a sensitive camera and cell lines expressing tagged proteins of interest. Due to advances in commercial microscopy platforms, CRISPR-based gene editing, and the widespread availability of organic fluorophores, these components are now routinely accessible within molecular biology departments. The primary barrier to the adoption of SMT is no longer the instrumentation or reagents but the complexity of data analysis and the challenge of translating SMT results into biological insight. Many different tracking and analysis tools have been developed by research groups across the world. These tools each perform specific aspects of downstream analysis of single molecule tracks and require reformatting of data or rewriting of code when switching from one program to another. This underscores the need for a self-contained software package that can not only track single molecules in timelapse images but also run multiple types of analyses. To make this accessible to the wider community we packaged this into a GUI, where each step can be performed with the click of a button. QuantiTrack comes with a detailed guide that walks the user through the set up and analysis, so that users without prior experience with MATLAB can carry out detailed analyses of their data efficiently. The data are stored in a convenient table format on which all the included analyses can be run, eliminating the need to reformat the data to fit the analysis workflow.

A simple GUI allows the user to load in a movie, detect and track particles, and perform quality control (Figures 1 and 2). Robust particle tracking is at the heart of SMT analysis. Given the sheer volume of data that an SMT experiment can generate, no individual can check every trajectory to ensure high tracking accuracy (although this has been done in at least one *tour de force* study^46^). To simplify this process and provide quantitative measurements of tracking errors, we include a dedicated Preprocessing panel (Figure 2) that generates QC metrics. This step must be performed before the tracks from an individual movie can be saved. This provides a quantitative measure for tracking errors and allows a user to empirically determine detection and tracking parameters (Figure 2). The post-processing panel allows the user to select from a rich palette of analysis options that can be run on the whole collection of tracks (Figure 1). These analyses quantify both the spatial and temporal dynamics of the protein of interest (with associated measures of error) and generate publication-quality figures and movies.

While not illustrated in this manuscript, QuantiTrack allows the user to draw multiple ROIs to track single molecules in the context of cellular features that are visualized using a different fluorescently labeled molecule: for example, an array of binding sites for a TF^27,47^, chromatin and chromatin marks^42,48,49^, or transcription sites^50^.

To demonstrate the utility of QuantiTrack, we applied it to quantify changes in GR dynamics in response to hormone treatment and washout. Even though the focus here has been on studying TF dynamics, QuantiTrack is by no means only a TF or nuclear protein tool since any SMT movie can be analyzed by QuantiTrack. The only difference would be that instead of histones, a different photobleaching probe would need to be selected for the quantification of lifetimes. Alternatively, analysis can be performed without photobleaching correction, but in this case any comparisons would be non-quantitative.

### GR binding and dynamics respond to changes in cellular hormone concentrations

Here, we used the glucocorticoid receptor to exemplify how QuantiTrack can serve as a simple yet effective tool to use SMT to uncover biological mechanisms. Our lab has pioneered studies on the cellular and molecular effects of ultradian hormone release on glucocorticoid signaling^21,23,24,26^. We have shown that hormone washout results in a loss of GR binding at a subset of GREs (as measured by chromatin immunoprecipitation [ChIP]^23,26^), by quantifying the accumulation of GFP-GR at a tandem array of GR-responsive MMTV promoters (through confocal microscopy), and by FRAP studies of GFP-GR at the MMTV array^23^. Here, using a combination of slow and fast SMT and multiple complementary analyses, we showed that throughout the nucleus, GR dynamics rapidly respond to changes in cellular hormone concentration as well. Hormone washout results in ~60% reduction in the GR bound fraction (Figure 3K), a significant decrease in GR dwell times (Figure 5D), the occupancy of GR in the lowest mobility state at the level of sub-tracks (which is correlated with active GR, Figure 5F) and full-length tracks (correlated with increased steroid receptor-mediated transcription, Figure 5I). These changes in GR binding and dynamics are subsequently reflected in the downregulation of GR target genes (Figure 3C).

GR is expressed in most cells of the human body and other vertebrates^51^. GCs are normally secreted in a pulsatile manner with a period of ~60–90 minutes. External stressors result in a spike in cortisol levels with chronic stress being associated with consistently elevated cortisol levels^25^. Our data are in an excellent agreement with the previously proposed Gene Pulsing model^23^ suggesting that GR can rapidly sense and respond to changes in cellular hormone concentrations. This is essential for the body to be able to distinguish between physiological GC pulses and GC release due to acute or chronic stress. GR’s sensitivity to cellular hormone concentrations likely evolved along with the patterns of GC release from the adrenal cortex, resulting in a feedback and feedforward loop linking upstream patterns of GC secretion to downstream changes in GR binding kinetics, in a process termed as continuous dynamic equilibration^25^.

The pulsatility of glucocorticoids and its effect on GR has tremendous implications for patients undergoing GC therapy^52^. A constant dose of GC mimics the physiological stress response, resulting in numerous side effects. Even the timing of a stressor relative to the physiological GC pulse determines the cellular stress response^53^. Thus, characterizing the effect of attenuated GC delivery patterns on GR dynamics is fundamental to devising appropriate treatment protocols. QuantiTrack provides the means to tackle this and many other questions in a robust, reproducible manner.

## LIMITATIONS

While developing QuantiTrack, we focused on creating a tool that would provide the most diverse information from a single program. As a result, several other analyses like vbSPT^54^, TrackIt^22^, state-array SPT^10^, among others have not been included here, but are freely available and can be incorporated into QuantiTrack by the user if so desired.

We chose to write this program in MATLAB since many existing MATLAB-based image analysis tools exist, which would lower the barrier-to-entry for a new user. However, MATLAB being a proprietary software, we do appreciate that not every user may have access to it.

An astute reader would have noticed that we use the term ‘bound’ and ‘stopped’ molecules interchangeably. We must emphasize that at this time, SMT does not have the resolution to identify site-specific binding events^8^. As a result, anything we call ‘bound’ refers to a spot that is stopped.

## METHODS

### Cell culture

MCF-7 cells (purchased from ATCC) were grown in Dulbecco’s Modified Eagle Medium (DMEM, Thermo Fisher Scientific # 10313039) supplemented with 10% fetal bovine serum (FBS, Gemini Bio, #900–308-500), 2 mM L-glutamine (Thermo Fisher Scientific #25030081), 1% MEM non-essential amino acids (Thermo Fisher Scientific #11140050), 1 mM sodium pyruvate (Thermo Fisher Scientific #11360070), and 50 U/mL penicillin and 50μg/mL streptomycin (Thermo Fisher Scientific #15070063).

### Generation of H2B-Halo and Halo-GR cell lines

To insert the HaloTag at the N-terminus of the *NR3C1* gene (NM_000176.3), which encodes the glucocorticoid receptor, gRNAs were cloned into the lentiCRISPR v2 (Addgene #52961) plasmid, which contains the Cas9 and puromycin resistance cassette (Figure S4A). This cell line was validated by Western blot (Figure S4B). GR was detected by chemiluminescence using the GR G-5 antibody (Santa Cruz Biotechnology # sc-393232).

To insert the HaloTag cassette at the C-terminus of the human *HIST1H2BG* gene (NM_003518), we designed a donor plasmid containing the HaloTag sequence flanked upstream by a homology arm (~800 bp) and 48 bp TEV linker, and downstream by a stop codon and a homology arm (~800 bp) (Figure S4C). These reagents have been previously validated for human cell lines^55^.

5×10^6^ MCF-7 cells at ~70% confluency were trypsinized and resuspended in 100μL of DMEM without FBS or antibiotics and electroporated with the respective donors and two gRNA-containing lentiCRISPR v2 plasmids. We used 2 gRNAs to improve the CRISPR efficiency. For the electroporation we prepared 5 million cells in 100μl of media (without FBS or antibiotics) with 5μg of each plasmid. We used the BTX T820 Electro Square Porator (Harvard Apparatus, Holliston, MA, USA) and applied 3× 10 ms pulses of 120 V. After electroporation, the cells were resuspended in complete fresh media. Since the cells transfected with the lentiCRISPR v2 plasmid acquire temporary resistance to puromycin, the electroporated cells were grown in the presence of 1μg/mL puromycin for two days. The surviving cells were washed three times with PBS and grown in medium without antibiotics until they reached 10×10^6^ cells.

Cells were then incubated with 5 nM Halo-TMR lig- and (Promega #G8251) for 20 min, washed three times, and the washed one more time after a 10 min incubation period. HaloTag expressing cells were then selected by fluorescence-activated cell sorting (FACS) and single positive clones were grown in 96 well plates. Several clones were selected and expanded further. Finally, we selected and validated one clone for each of the cell lines for this study.

### RNA-qPCR experiments

For RNA-qPCR experiments, MCF-7 cells were plated in 6 well plates in medium containing charcoal-stripped FBS for 24 hours. In the treatment protocol, cells were treated with 1.2μM Hcort (Sigma Aldrich #H4001–1G) for 20 min or 60 min. In the washout protocol, MCF-7 cells were treated with 1.2μM Hcort for 20 min and then washed three times with conditioned medium. RNA was collected at 20 min and 60 min using the PureLink RNA Mini Kit from Thermo Fisher Scientific (Catalog number 12183025) following the manufacturer’s protocol. For the control samples, cells were treated with 0.1% EtOH for 60 min, and this serves as our baseline.

### Single molecule tracking sample preparation

100,000 MCF-7 cells expressing Halo-GR or H2B-Halo were plated in 2 well chamber slides (Cellvis #C2–1.5H-N) in charcoal-stripped FBS supplemented medium for 24 hrs. On the day of imaging, cells were incubated with 50 pM JF549 (Promega # GA1110; for the slow SMT experiments) or 100 pM JFX650 (Promega #HT1070; fast SMT experiments) for 15 min. The samples were washed three times with phenol red-free charcoal-stripped FBS containing medium. The samples were returned to the incubator for 10 min and washed one more time.

After the dye labeling step was complete, the samples were treated with 1.2μM Hcort for 20 min. For the washout condition, the sample was washed three times with phenol red-free charcoal-stripped FBS medium, while nothing further was done to the samples in the treatment protocol. The chamber slides were then allowed to equilibrate on the microscope for 10 min before collecting movies. Data were collected between 30 and 60 min after initial hormone addition.

### Microscopy

All the data reported in this study were collected on a custom-built highly inclined and laminated optical sheet microscope at the Optical Microscopy Core of the NCI. This system has been described and characterized in detail in a previous publication^56^. Briefly, this microscope is built on the body of an Olympus IX81 inverted microscope and is equipped with a 150×, 1.45 NA objective (Olympus Scientific Solutions, Waltham, MA, USA). This system has a 561 nm laser (iFLEX-Mustang, Excelitas Technologies Corp., Waltham, MA, USA), a 647 nm laser (OBIS 647 LX, Coherent Inc, Santa Clara, CA, USA), an acousto-optical tunable filter (AOTFnC-400.650, AA Optoelectronic, Orsay, France), and an EM-CCD camera (Evolve 512, Photo-metrics, Tucson, AZ, USA). For live cell-imaging, the microscope has a stage-top incubator with 5% CO_2_ control (Okolab, Pozzuoli NA, Italy).

For fast SMT experiments, images were collected every 12 ms under continuous illumination by the 647 nm laser (Figure 3D). For slow SMT experiments, movies were collected under stroboscopic illumination by the 561 nm laser with 10 ms exposures at a frame rate of 5 Hz (200 ms intervals, with a 190 ms dark period between consecutive illuminations) (Figure 5A). Both laser powers were set to 0.96 mW. The pixel size for this setup is 104 nm/pixel.

### Tracking

Image stacks were filtered using a bandpass filter. The nuclear periphery was defined using a hand-drawn region of interest based on the sum projection of the stack. The Threshold parameter was set such that less than 5% of detected particles had SNR < 1.5. Particles were localized by fitting a Gaussian point spread function. Detected particles were connected into tracks based on a nearest neighbor algorithm. The maximum jump parameter was optimized based on the second nearest neighbor histogram (see Quality control). Given that previous FRAP and FCS measurements have found that GR has a free diffusion constant of $\sim 5\;{\mu \text{m}}^{2}/\text{s}$^39,57–59^, freely diffusing GR molecules can move up to 600 nm in 12 ms, corresponding to a 6 pixel max jump size. On the other hand, bound molecules with a diffusion constant of $\sim 0.1\;{\mu \text{m}}^{2}/s$ will move on the order of 400 nm over 200 ms, corresponding to a max jump of 4 pixels. Thus, the following tracking parameters were used: for fast SMT, max jump = 6 pixels, gaps to close = 1 frame, shortest track = 2 frames, while for slow SMT, max jump = 4 pixels, gaps to close = 1 frame, shortest track = 2 frames.

### Quality control

We developed two types of quality control measures that can be applied to each movie while it is being analyzed, or later – on an ensemble of movies. We describe these below:

#### Signal-to-noise ratio

For every detected particle, the particle intensity is calculated as the average intensity over the Airy disk, which has a radius (in pixels), $r_{\text{Airy}}$ given by $r_{\text{Airy}}=\frac{1.22\lambda }{2\rho NA}$, where λ is the wavelength of the emitted light, ρ is the pixel size and *NA* is the numerical aperture of the objective. The local background is calculated as the mean intensity over a donut-shaped region with inner and outer radii (in pixels) calculated as $r_{\text{inner}}=r_{\text{Airy}}+2$ and $r_{\text{outer}}=r_{\text{Airy}}+3$ respectively (Figure 2C, inset).

The signal-to-noise ratio (SNR) for each particle is then calculated as

$$\text{SNR}=\frac{I_{\text{particle}}-I_{\text{background}}}{\sqrt{I_{\text{background}}}}$$

where $I_{\text{particle}}$ and $I_{\text{background}}$ are the particle and local background intensity respectively.

Throughout the datasets analyzed in this study, we ensure that less than 5% of all detected particles have an SNR lower than 1.5. This provides an objective criterion for a user to gauge whether they are detecting bona fide particles or simply detecting background.

#### Distance to second nearest neighbor

Mistracking could occur due to dense labeling or due to the maximum jump parameter being set too high. In this scenario, a particle in frame n could be incorrectly linked to a different particle in frame n+1 (Figure 2F, inset). To quantify the frequency of such errors, we calculate the distance between a tracked particle and its second nearest neighbor in the subsequent frame. We ensure that the tracking parameters are selected such that the fraction of second nearest neighbors within the maximum jump distance is less than 5%.

### Localization precision

The precision with which we estimate the position of each particle is calculated from the standard deviations ($\sigma_{x}$ and $\sigma_{y}$) of the fitted coordinates, which are combined to get $\sigma_{\text{total}}=\sqrt{\sigma_{x}^{2}+\sigma_{y}^{2}}$. For each image QuantiTrack reports the median $\sigma_{\text{total}}$ from all detected particles.

### Dwell time distribution

To identify bound segments within a track, the user must determine the distance a stably bound molecule moves over consecutive frames $\left(r_{\text{min}}\right)$. Since transcription factors interact with chromatin, we used histone H2B as our reference molecule, and the parameter $r_{\text{min}}$ is calculated as the 99^th^ percentile of the H2B frame-toframe displacements. Freely diffusing molecules can move less than this threshold distance in one frame, but the probability that a freely diffusing molecule with diffusion coefficient $D_{\text{free}}$, moves a distance $r_{\text{min}}$ over each of $N_{\text{min}}$ frames in time Δt is given by

$$\text{P}\left({\text{r}}_{\text{min}},{\text{N}}_{\text{min}}\right)={\left(1-\text{exp}\left(\frac{{\text{r}}_{\text{min}}^{2}}{4{\text{D}}_{\text{free}}\Delta \text{t}}\right)\right)}^{N_{\text{min}}}$$

We used this equation to determine the number of frames $N_{\text{min}}$ over which the probability of classifying a diffusing molecule as bound is less than 1%. We then determined the distance $r_{\text{max}}$, which is the 99^th^ percentile of the H2B displacement histogram over $N_{\text{min}}$ frames, and further eliminated track segments where the TF displacement was larger than $r_{\text{max}}$ over $N_{\text{min}}$ frames. A survival distribution was then compiled from all the bound segments.

### Photobleaching correction

The histone H2B survival distribution was used to measure the photobleaching rate and correct for photobleaching using our previously described method^21^. We generated the survival distribution for H2B as described in the section ‘Dwell time distribution’. We fit this survival distribution to a triple exponential function and extracted the slowest exponential decay rate as the photobleaching rate $k_{PB}$. The raw survival distribution $S_{\text{raw}}(t)$ is then corrected for photobleaching as follows, where $t_{0}$ is the first point of the survival distribution:

$$S_{\text{corrected}}(t)=\frac{S_{\text{raw}}(t)}{e^{-k_{PB}\left(t-t_{0}\right)}}$$

Finally, we normalize the survival distribution to the bound fraction such that:

$$S_{\text{corrected}}^{\text{norm}}(t)=S_{\text{corrected}}(t)\times F_{\text{bound}}$$

### RL analysis

The self-part of the jump distance histogram or van Hove correlation (vHc) was calculated as $G_{s}(r,\tau )=A_{s}\left\langle \delta \left(r_{i}-\left|r_{i}(t+\tau )-r_{i}(t)\right|\right\rangle \right.$, where $r_{i}$ is the position of the i^th^ particle and $A_{s}=\int d^{2}rG_{s}(r,\tau )$ is a normalization constant. As done previously^11,13^, we assumed that the vHc can be expanded as a superposition of Gaussian functions $q(r,M)=\left(\frac{1}{\pi M}\right)\text{exp}\left(-\frac{r^{2}}{M}\right)$ such that $G_{S}(r,\tau )=\int P(M,\tau )q(r,M)dM$, where M is the mean-squared displacement and P(M) is the probability density of the mean-squared displacements of the ensemble of particles. We then used an iterative scheme developed by Richardson and Lucy and recently adapted to nucleosomes^13^ and other transcriptional proteins^11^. From an initial distribution $P^{0}(M)=\text{exp}\left(-\frac{M}{M_{0}}\right)$, the distribution in the $(n+1{)}^{th}$ iteration, $P^{n+1}$, can be written as

$$P^{n+1}=P^{n}\int \frac{G_{s}\left(r,\tau \right)}{{G^{n}}_{s}\left(r,\tau \right)}q\left(r,M\right)d^{2}r$$

Since $P^{n+1}$ is a probability density, we impose the constraint $P^{n+1}>0$ and that $P^{n+1}$ be normalized.

To classify into different mobility groups, we first identified the local minima in the inferred MSD distributions (Figures 4B, D, and 5H). We used these local minima to bin the MSD distribution into different mobility groups. Each track was assigned to the bin corresponding to its MSD at the specified time lag as previously described^11,13,60^.

### Perturbation expectation maximization v2 analysis

Perturbation expectation maximization v2^45^ was run as described previously^11^. Briefly, all tracks were divided into 7-frame sub-tracks, thus retaining only those tracks that are at least 7 frames long. We chose a split length of 7 frames to minimize transitions between different mobility states within a single sub-track. Different split lengths have been tested previously, yielding similar results^27^. The system was allowed to explore between 1 and 15 states with 50 reinitializations and 200 perturbations. Up to 10,000 iterations were allowed and the convergence criteria for the log-likelihood function was set at 10^−7^. Convergence of pEMv2 was verified by running each dataset five times, and the run with the lowest BIC was selected for further analysis.

The output from pEMv2 is the optimal number of states and a posterior probability for each sub-track. We assigned the sub-tracks to the states for which they had the highest posterior probability (Figure S10A). For MSD visualization and population fraction calculations, we only considered those sub-tracks for which the difference between the two highest posterior probabilities was greater than 0.2. This was done to ensure that sub-tracks with similar posterior probabilities for multiple states do not contaminate the data.

### Angle anisotropy analysis

Angles formed by 3 successive localizations were calculated for all tracks and plotted as a polar histogram. As previously described by Izeddin, et al^20^, we calculated the anisotropy coefficient (AC) as the log_2_ ratio of the probability of forward displacements $\left[\text{prob}\left(-30^{{}^{\circ}}\leq \theta \leq 30^{{}^{\circ}}\right)\right]$ to that of backward displacements $\left[\text{prob}\left(150^{{}^{\circ}}\leq \theta \leq 210^{{}^{\circ}}\right)\right]$ as follows (Figure 4E):


$$\text{Anisotropy}\;\text{coefficient}={\text{log}}_{2}\left(\frac{\text{prob}\left(-30{}^{\circ}\leq \theta \leq 30{}^{\circ}\right)}{\text{prob}\left(150{}^{\circ}\leq \theta \leq 210{}^{\circ}\right)}\right)$$

The spatial anisotropy curve is obtained by binning the angles obtained at all lag times based on the average displacement of the consecutive jumps and then calculating AC for each binned distance.

### Bound fraction

#### Full kinetic model

To obtain a good estimate of the bound fraction, we use a previously described method to simultaneously fit the jump histograms for multiple time lags to the expected histograms output from one or more kinetic models^19^. All data presented here were fitted with a model that included one freely diffusive population and one bound population. The free parameters of the model include the association and dissociation rate constants, k_ON_ and k_OFF_ respectively, which are used to calculate the bound fraction, $F_{\text{bound}}=\frac{k_{\text{ON}}}{\left(k_{\text{ON}}+k_{\text{OFF}}\right)}$. Errors in the fit were estimated as the standard deviation in the fits of the jump histograms from 100 bootstrapped samples with replacement.

#### Spot-On

Spot-On was run with the indicated time lags with the following parameters:

TimeGap = 12; dZ = 0.7; GapsAllowed = 1; TimePoints = 7; BinWidth = 0.01; UseEntireTraj = 1; UseWeights = 1; MaxJump = 0.7 (GR + Hcort treatment). MaxJump = 1.5 (GR + Hcort washout); Model-Fit = 1; DoSingleCellFit = 0; FitIterations = 5; Fit-LocError = 1; FitLocErrorRange = [0.01, 0.075]; NumberOfStates = 2; D_Free_2State = [0.05, 75]; D_Bound_2State = [1e-5, 0.05].

Error bars represent the standard deviation of 100 bootstrapped samples with replacement.

## Supplementary Material

Supplement 1

Supplement 2

Supplement 3

Supplement 4

## Data Availability

QuantiTrack and all the SMT data used in this study are deposited in Zenodo (https://doi.org/10.5281/ze-nodo.18562861).
