# Supplementary material for: QuantiTrack: A unified software to study protein dynamics in living cells
Source: bioRxiv. 2026 Feb 27:2026.02.19.706877. Originally published 2026 Feb 20. Preprint. [Version 2] doi: 10.64898/2026.02.19.706877 (PMC12934656; doi:10.64898/2026.02.19.706877)
Supplement: Supplement 1 [file media-1.pdf]

QUANTITRACK: AN INTEGRATED GUI-BASED SINGLE-MOLECULE TRACKING AND ANALYSIS TOOL

David A Ball†, Kaustubh Wagh†, Diana A Stavreva, Le Hoang, R Louis Schiltz, Raj Chari, Razi Raziuddin, Davide Mazza, Arpita Upadhyaya, Gordon L Hager*, Tatiana S Karpova*

†Co-first authors

*Co-corresponding authors

SUPPLEMENTARY MATERIALS

This PDF includes

Figures S1 to S10

Movies 1 to 3

Supplementary Figure 1

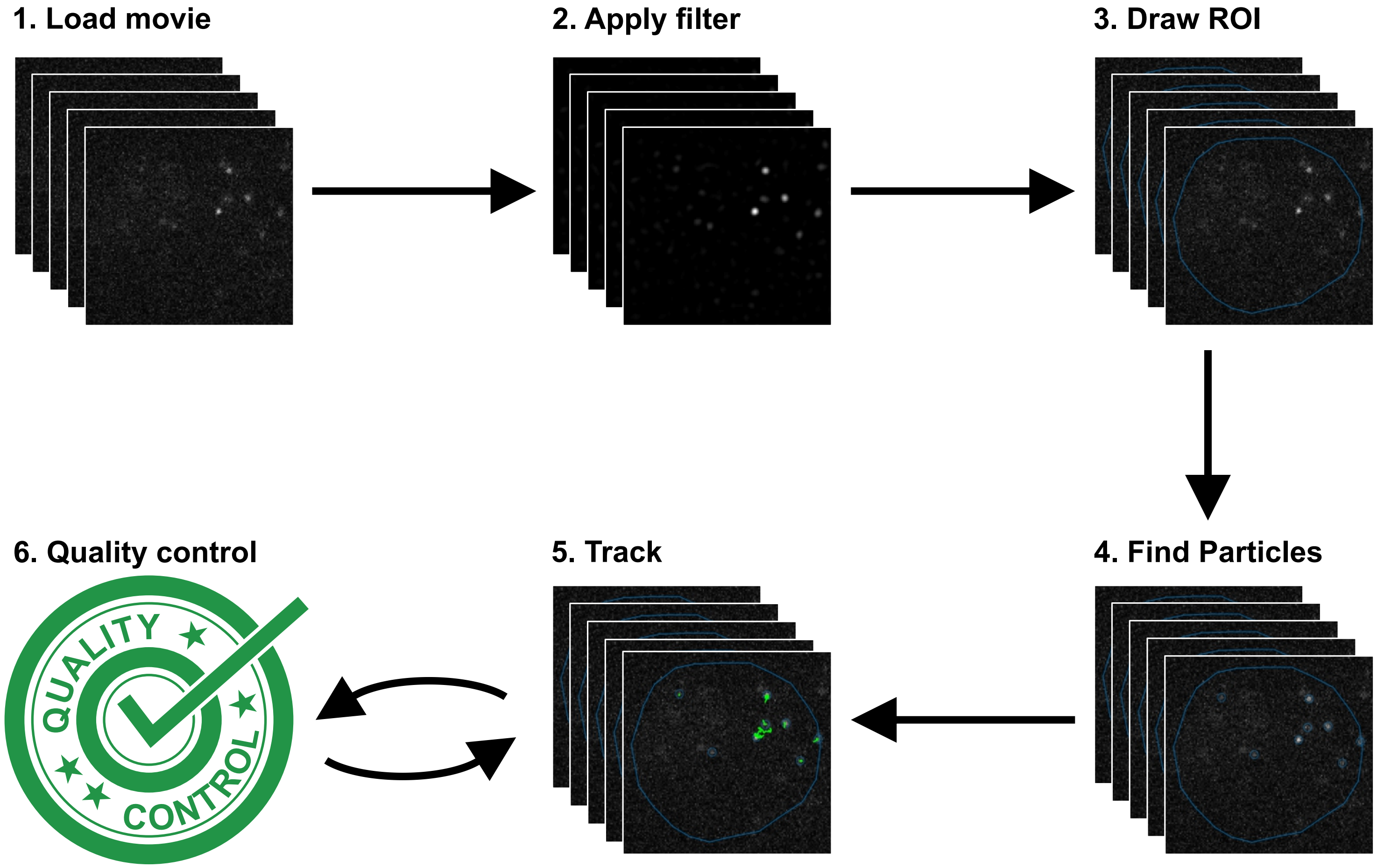

Supplementary Figure 1: QuantiTrack workflow.

Schematic of the QuantiTrack workflow. (1) Load the tif stack into QuantiTrack. (2) Filter the movie using Bandpass, Laplacian of Gaussian (LoG), or local background filters. (3) Draw a region of interest (ROI) to demarcate the nucleus or other features of interest. (4) Find particles. (5) Combine detected particles into tracks. (6) Run the quality control analysis and update tracking or particle detection parameters if needed.

Supplementary Figure 2

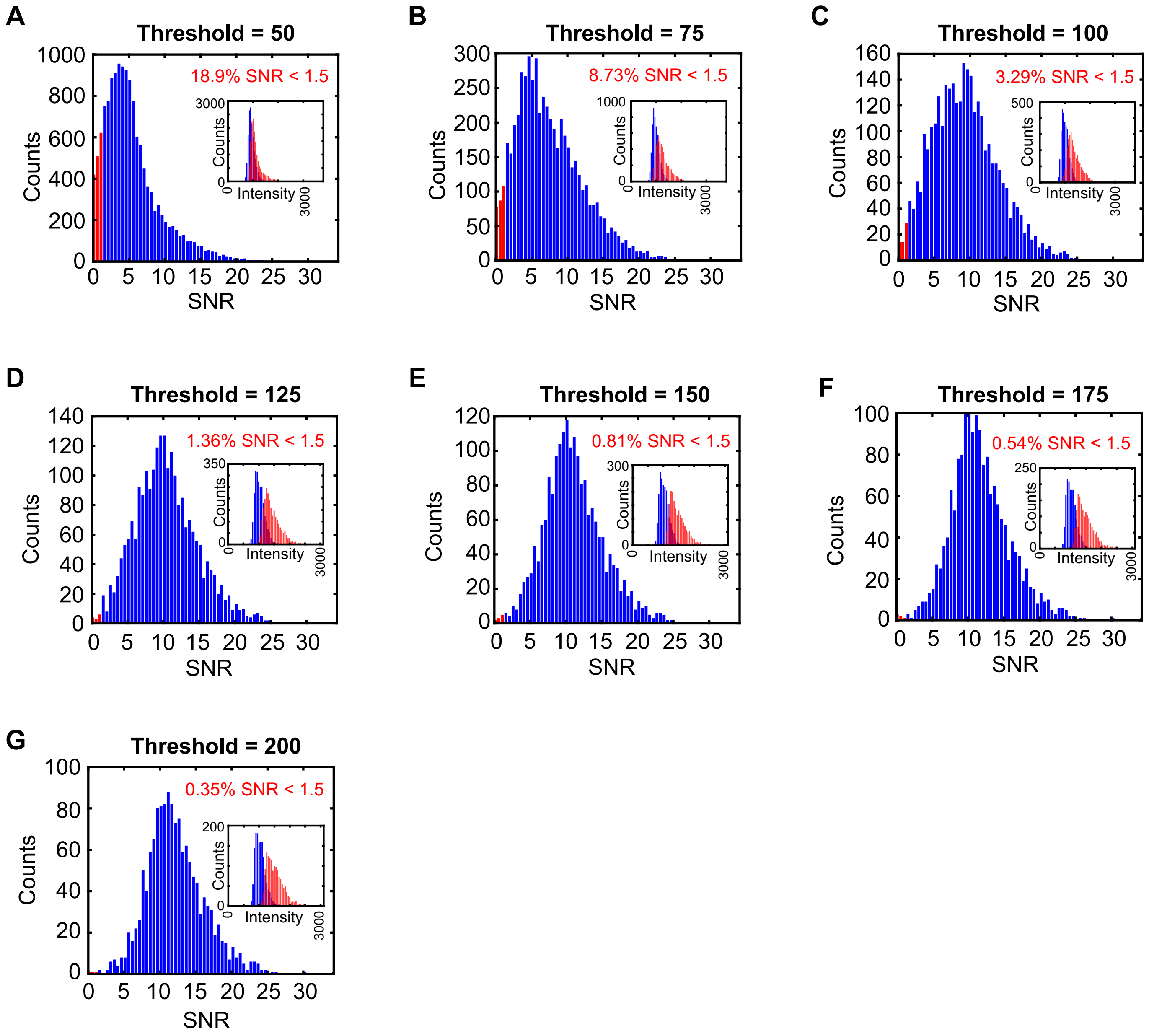

Supplementary Figure 2: Signal-to-noise ratio (SNR) histograms for Threshold parameter sweep.

Histogram of SNR for the indicated Threshold parameter values. The histogram bins in red are those with SNR < 1.5. (Inset) Histogram of particle intensity (red) and local background (blue).

Supplementary Figure 3

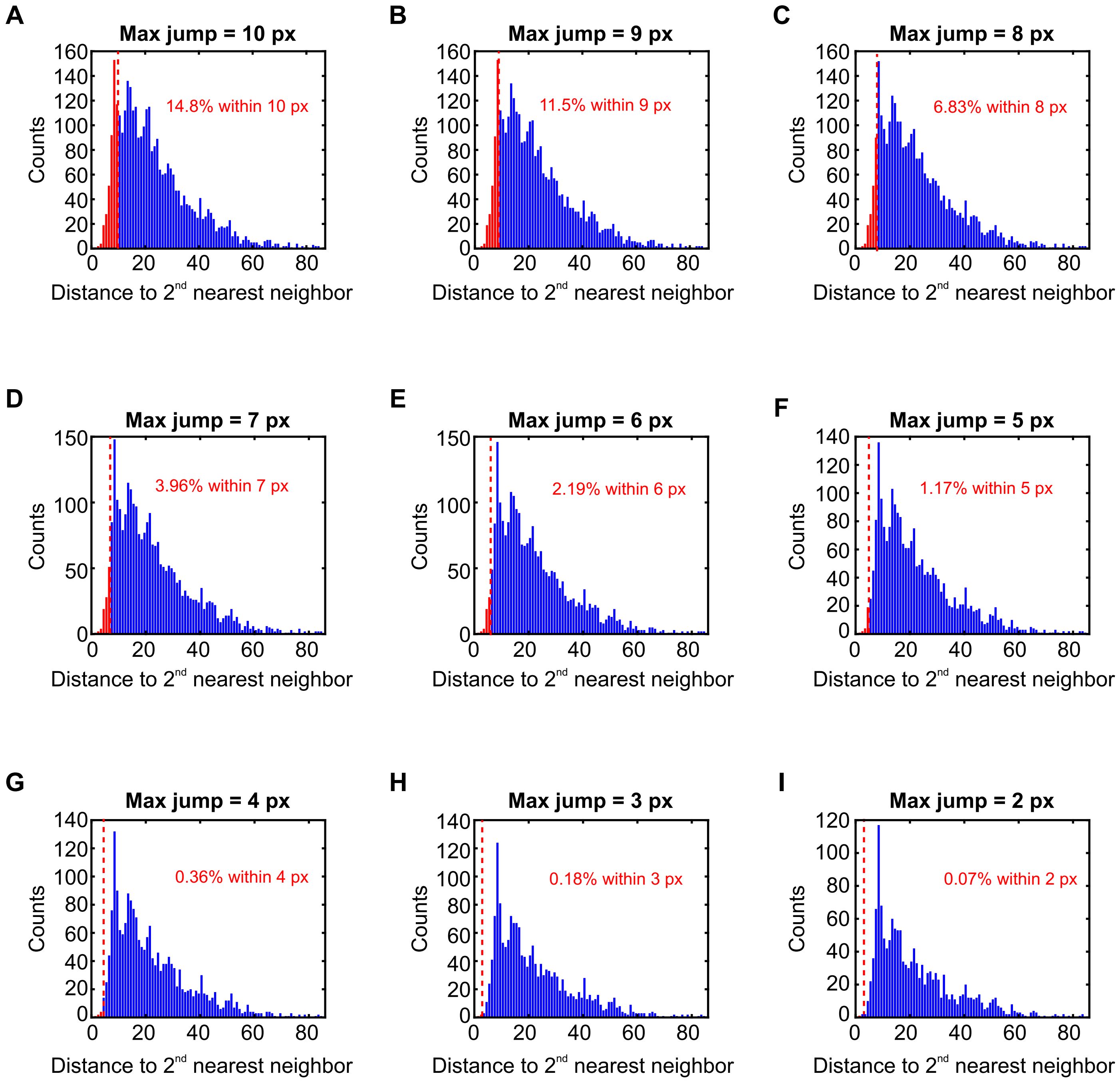

Supplementary Figure 3: Second nearest neighbor distance histograms for maximum jump parameter sweep.

Histogram of the distance between a particle and its second nearest neighbor in the subsequent frame for the indicated maximum jump parameter values. The histogram bins in red are those smaller than the maximum jump value. The inset text denotes the fraction of particles with second nearest neighbors within the respective maximum jump.

Supplementary Figure 4

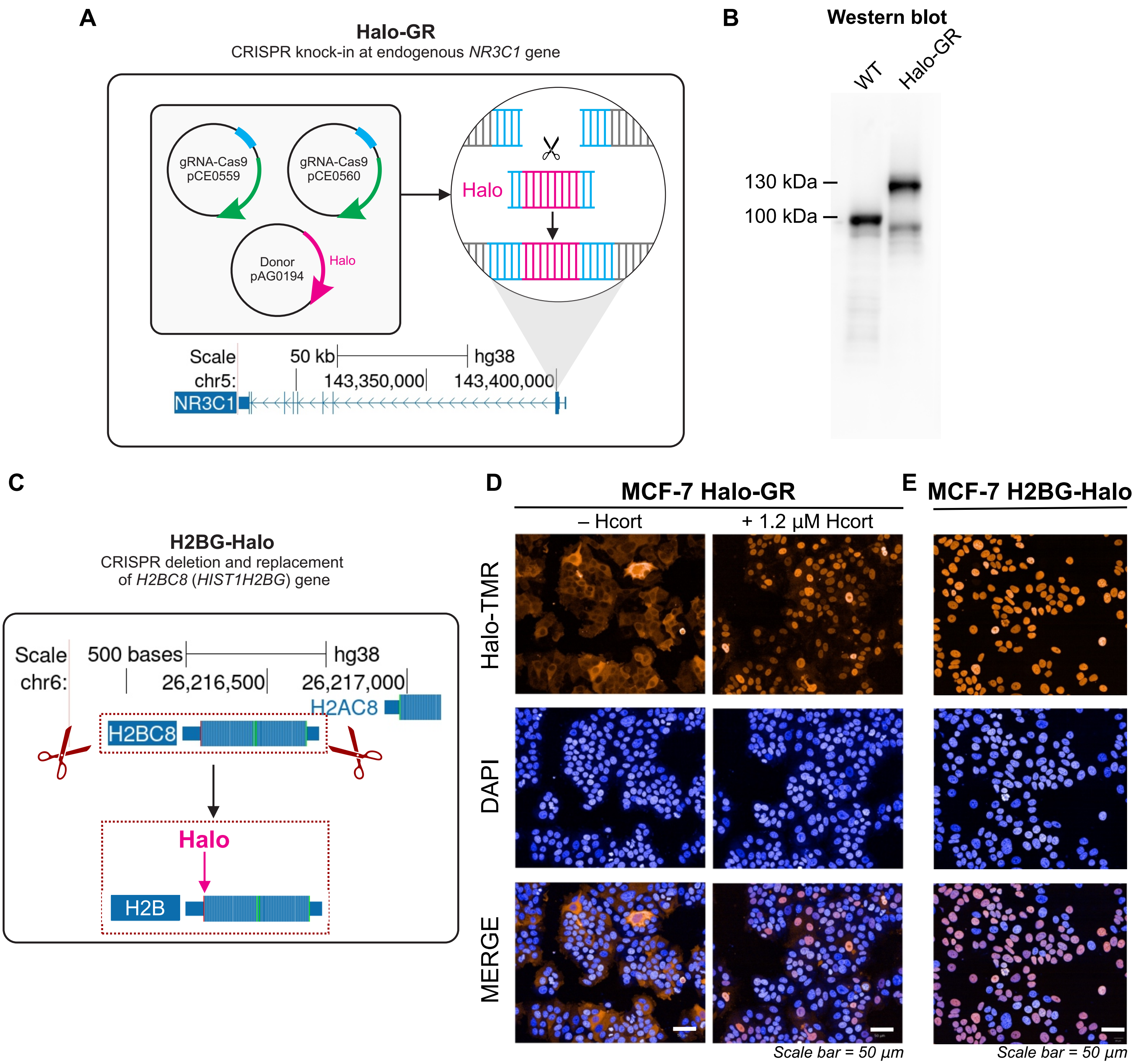

Supplementary Figure 4: Generation and validation of Halo-GR and H2B-Halo MCF-7 cell lines.

(A) To tag the endogenous GR in MCF-7 cells, the HaloTag cassette was inserted at the 5' end of the *NR3C1* gene (which encodes the glucocorticoid receptor [GR]) using CRISPR-Cas9. **(B)** Western blot for GR in the parental MCF-7 cells (WT) and clonal Halo-GR CRISPR knock-in cell line (Halo-GR). Expected sizes: 97 kDa (GR), 130 kDa (Halo-GR). **(C)** To generate the H2B-Halo cell line, the *H2BC8* gene was excised and replaced with an *H2B-HALO* gene using CRISPR-Cas9, and is expressed from the endogenous promoter. **(D)** Representative images of MCF-7 cells expressing Halo-GR. Halo-TMR staining (which covalently binds to the HaloTag) is shown in orange and DAPI staining is shown in blue. The column on the left shows the cells before hormone treatment, where GR is primarily cytoplasmic. The column on the right shows GR after treatment with 1.2 μM hydrocortisone (Hcort), which results in nuclear translocation of GR. **(E)** Representative images of H2B-Halo expressing MCF-7 cells. Halo-TMR staining is shown in orange while DAPI staining is shown in blue. Scale bars for panels D and E are 50 μm.

Supplementary Figure 5

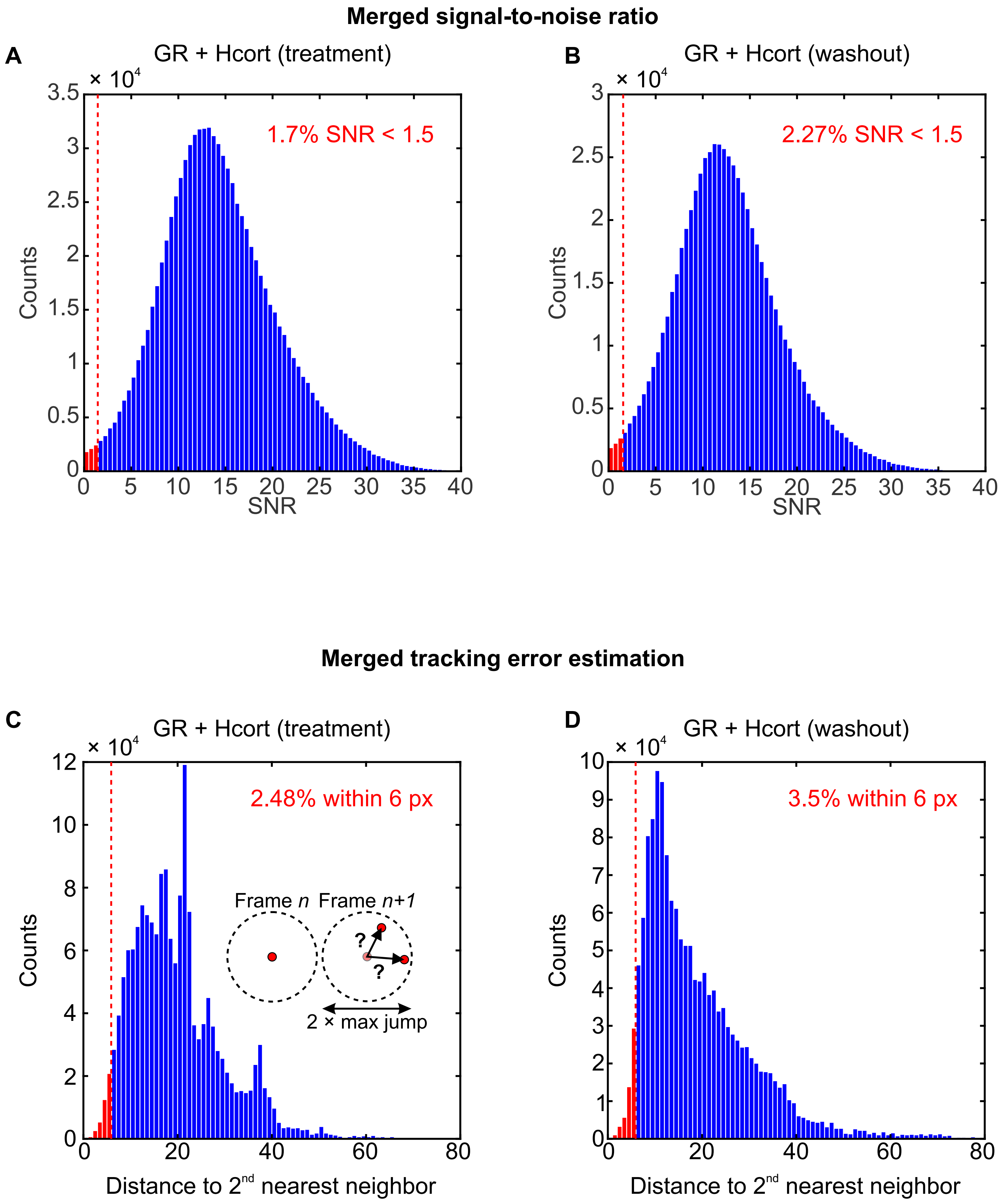

Supplementary Figure 5: Merged quality control metrics for fast SMT.

(A–B) Histograms of signal-to-noise ratios (SNR) for all detected particles. (A) GR + Hcort treatment, (B) GR + Hcort washout. Bins with SNR < 1.5 are shown in red. Insets show the percentages of particles with SNR < 1.5. **(C–D)** Histogram of distances between a tracked particle in frame *n* and the second nearest neighbor in frame *n+1* for (C) GR + Hcort treatment, and (D) GR + Hcort washout. Bins with distances ≤ max jump are depicted in red. The inset shows the percentage of second nearest neighbor distances within the max jump radius.

Supplementary Figure 6

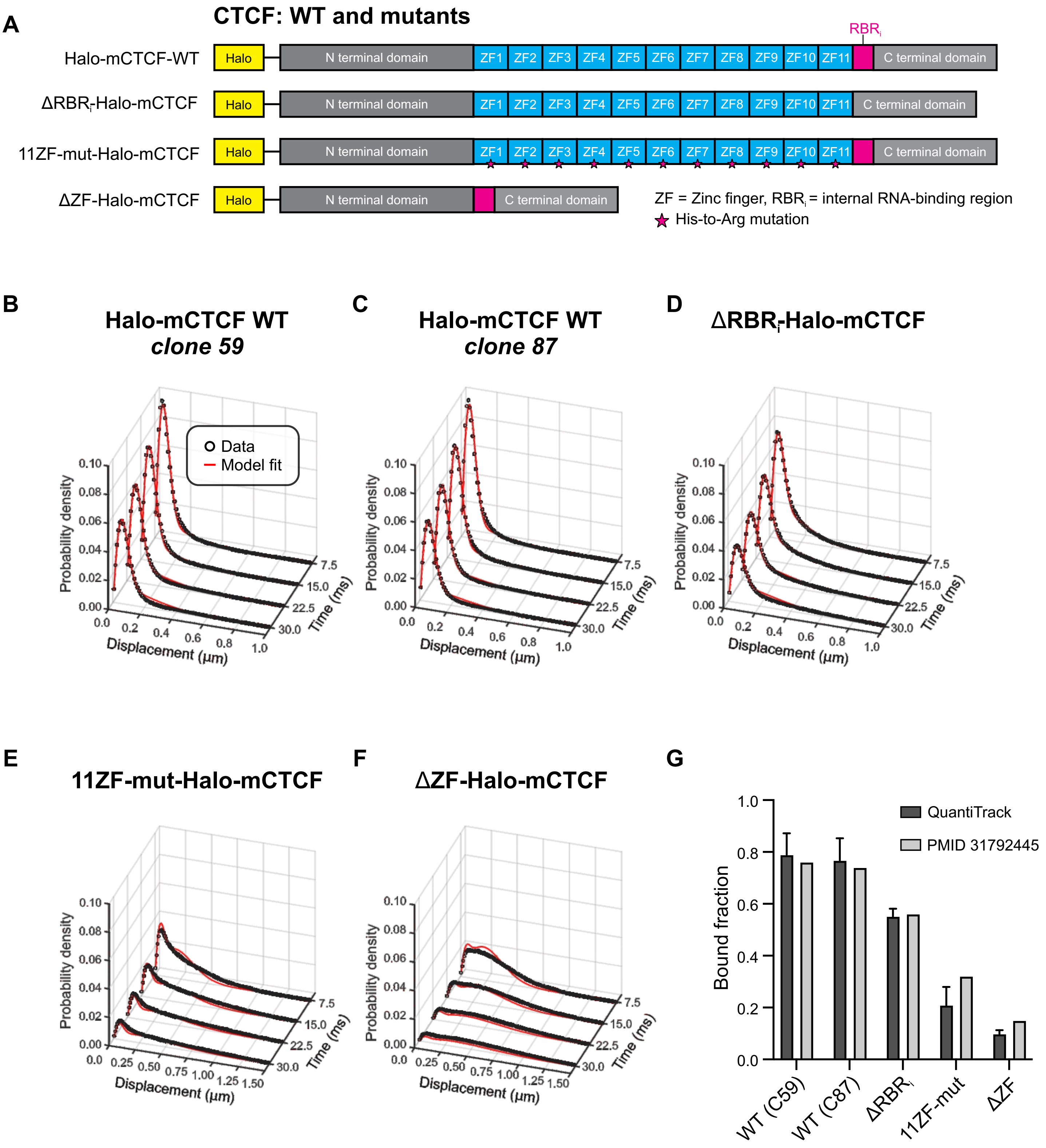

Supplementary Figure 6: Comparison of QuantiTrack and Spot-On on publicly available data.

(A) CTCF contains an N-terminal domain, 11 zinc fingers (ZF), an internal RNA-binding domain (RBR_i), and a C-terminal domain. Here we examined the bound fraction of wildtype CTCF (Halo-mCTCF-WT), a CTCF mutant lacking RBR_i (ΔRBR_i-Halo-mCTCF), a CTCF mutant with a His-to-Arg mutation in all 11 ZFs (11ZF-mut-Halo-mCTCF), and a CTCF mutant with all the ZFs deleted (ΔZF-Halo-mCTCF) in mouse embryonic stem cells. **(B–F)** Kinetic modeling of jump distance histograms over four time lags using a two state (bound + diffusive) model for (B) endogenous Halo-mCTCF (clone 59), (C) endogenous Halo-mCTCF (clone 87), (D) ΔRBR_i-Halo-mCTCF, (E) 11ZF-mut-Halo-mCTCF, and (F) ΔZF-Halo-mCTCF. The data are shown as black circles and the model fit is in red. **(G)** Bound fraction calculated from the kinetic model (dark gray), and those reported by Hansen et al [40] (light gray). Error bars = bootstrapped standard deviation.

Supplementary Figure 7

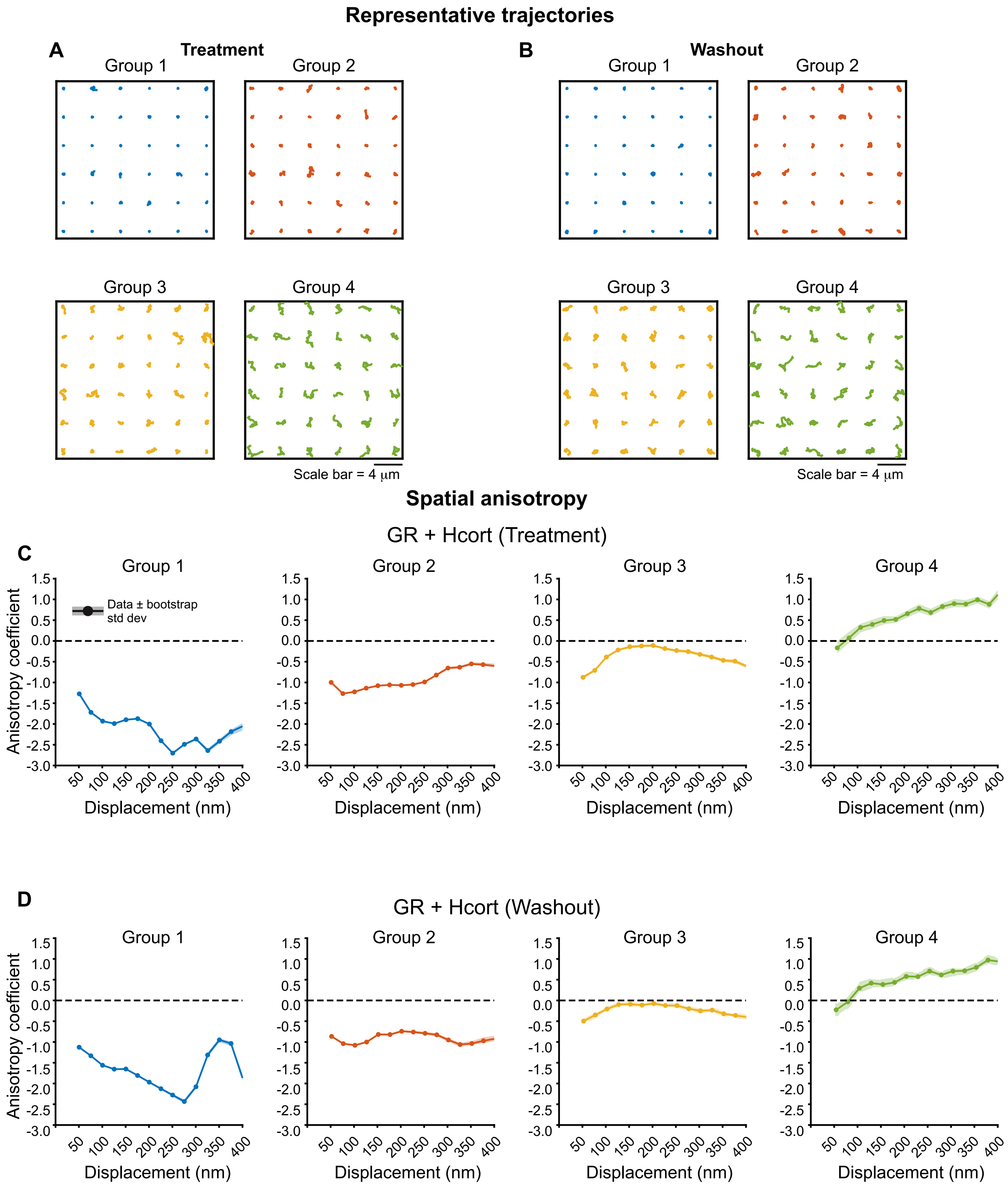

Supplementary Figure 7: Representative trajectories and spatial anisotropy for different RL groups detected in fast SMT.

(A–B) 36 randomly selected trajectories belonging to the indicated RL groups for (A) GR + Hcort treatment and (B) GR + Hcort washout. Scale bar = 4 μm. **(C–D)** Spatial anisotropy (i.e. the anisotropy coefficient calculated as a function of the average displacement of the two vectors forming the angle) of the indicated RL groups for (C) GR + Hcort treatment and (D) washout. Shaded error bars = bootstrapped standard deviation.

Supplementary Figure 8

A

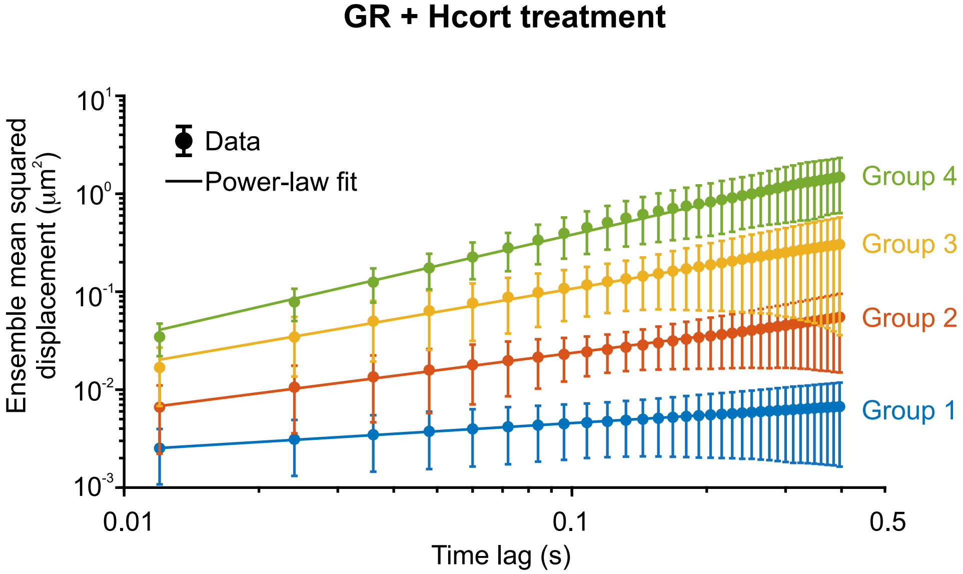

B

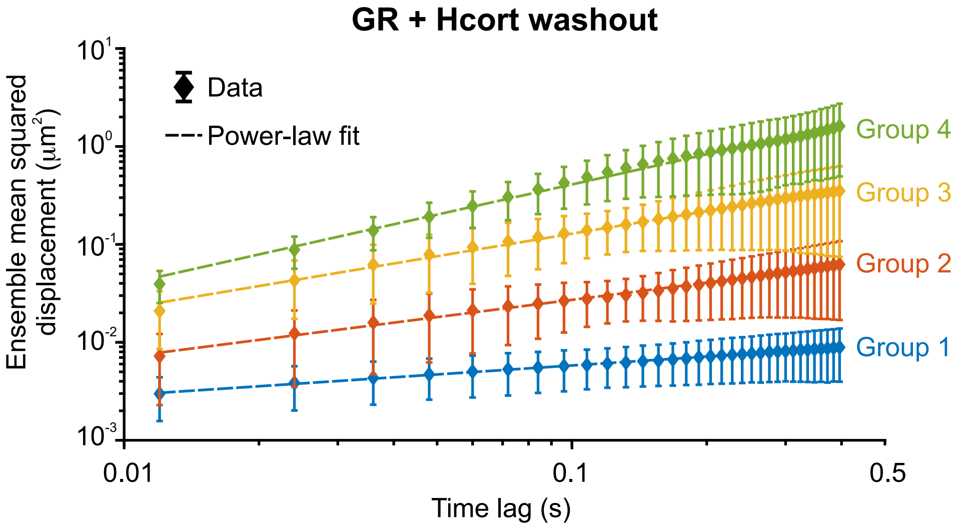

Supplementary Figure 8: Ensemble mean-squared displacement for different RL groups.

(A–B) The ensemble mean-squared displacement for GR after (A) Hcort treatment and (B) washout for each RL group. Power-law fits are shown as solid lines for GR + Hcort treatment and as dashed lines for GR + Hcort washout. Error bars = standard deviation.

Supplementary Figure 9

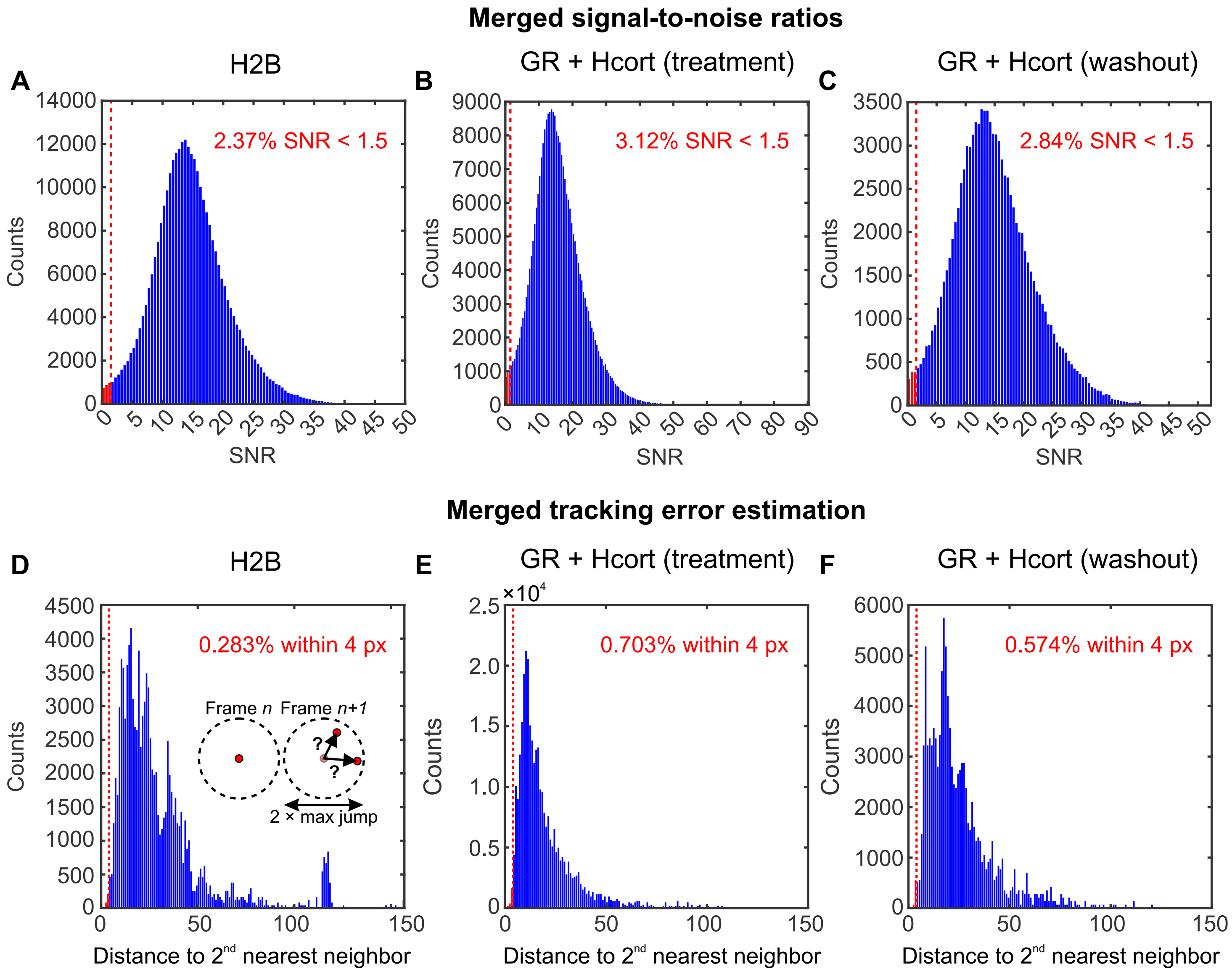

Supplementary Figure 9: Merged quality control metrics for slow SMT.

(A–C) Histograms of signal-to-noise ratios (SNR) for all detected particles. (A) H2B, (B) GR with hydrocortisone (Hcort) treatment, (C) GR with Hcort washout. Bins with SNR < 1.5 are shown in red. Insets show the percentages of particles with SNR < 1.5. **(D–F)** Histogram of the distance between a tracked particle in frame *n* and the second nearest neighbor in frame *n+1* for (D) H2B, (E) GR under Hcort treatment, and (F) GR under Hcort washout. Bins with distance ≤ max jump are depicted in red. The inset shows the percentage of second nearest neighbor distances within the max jump radius.

Supplementary Figure 10

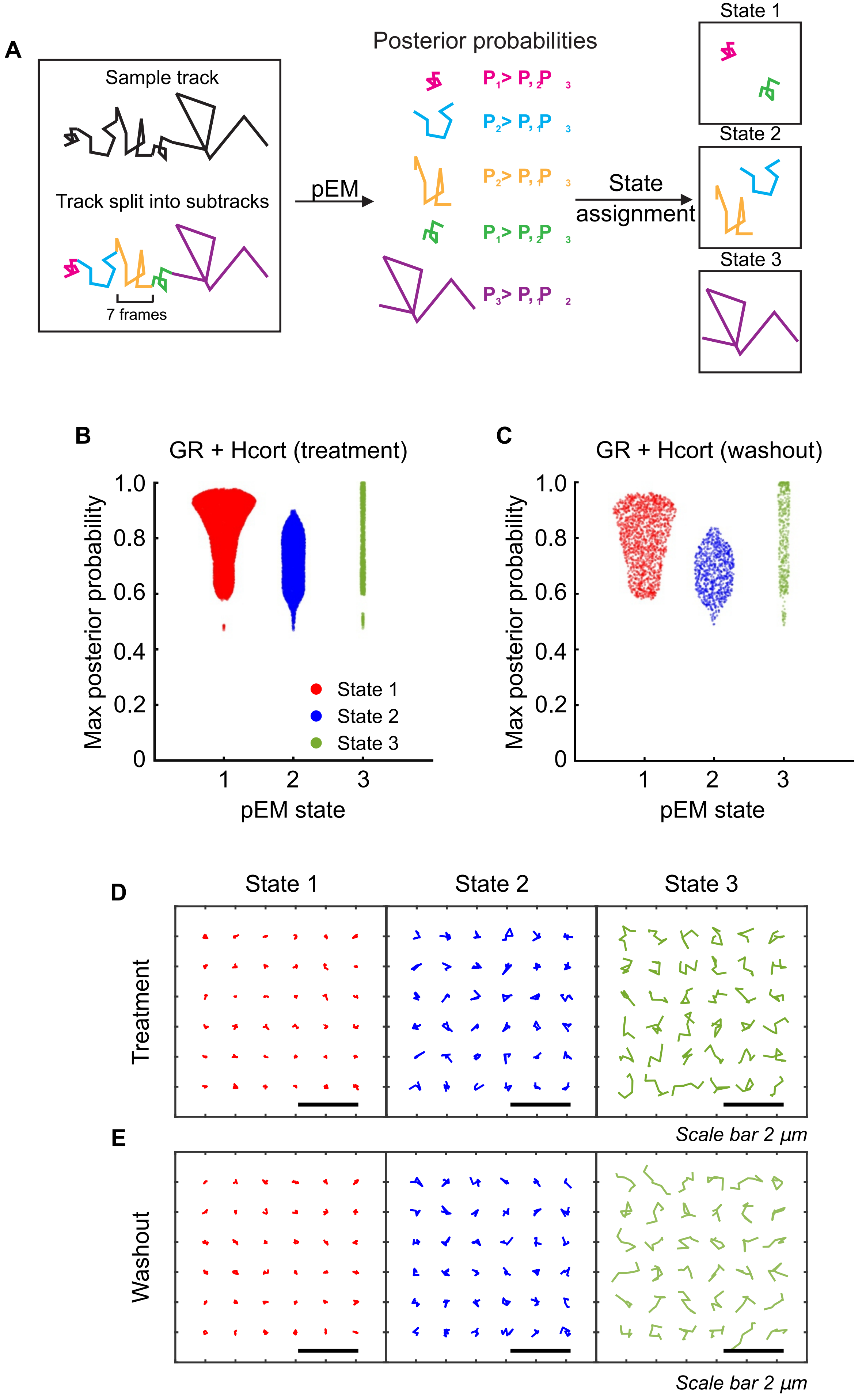

Supplementary Figure 10: Perturbation-expectation maximization.

(A) Schematic of the perturbation-expectation maximization (pEMv2) workflow. (Left) Tracks are split into 7-frame sub-tracks. (Center) After running the pEMv2 analysis, each sub-track has a posterior probability of belonging to each mobility state. In this schematic, pEMv2 converged to 3 states. (Right) Each sub-track is assigned to the state for which it has the highest posterior probability. **(B–C)** Swam charts of the maximum posterior probability for each pEMv2 state for GR under (B) Hcort treatment and (C) Hcort washout. **(D–E)** 36 randomly selected sub-tracks assigned to state 1 (red, left), state 2 (blue, middle), and state 3 (green, right) for GR under (D) Hcort treatment and (E) Hcort washout. Scale bar = 2 μm. Panel A was re-used from [11] (public domain).

Movie 1

(2+1) D movie of GR + Hcort obtained with 12 ms exposures and continuous illumination.

Movie 2

Fast SMT movie of Halo-GR + Hcort treatment (left) and washout (right) overlaid with detected tracks (red). Only tracks longer than 10 frames are shown. Scale bar = 2 μm.

Movie 3

Slow SMT movie of H2B-Halo (left), Halo-GR + Hcort treatment (middle), Halo-GR + Hcort washout (right) overlaid with detected tracks (red). Only tracks longer than 10 frames are shown. Scale bar = 2 μm.
